# CauFinder: Steering Cell‐State and Phenotype Transitions by Causal Disentanglement Learning

**DOI:** 10.1002/advs.76177

**Published:** 2026-06-16

**Authors:** Chengming Zhang, Zexi Chen, Yuanxiang Miao, Zuolin Shen, Deyu Cai, Shijie Tang, Yun Xue, Weifeng Guo, Hongbin Ji, Jian Liu, Kazuyuki Aihara, Luonan Chen

**Affiliations:** ^1^ International Research Center For Neurointelligence, The University of Tokyo Institutes For Advanced Study The University of Tokyo Tokyo Japan; ^2^ Department of Medical Oncology, Shanghai Pulmonary Hospital Tongji University School of Medicine Shanghai China; ^3^ School of Life Science and Technology ShanghaiTech University Shanghai China; ^4^ Centre for Infection Immunity and Cancer (IIC) of Zhejiang University‐University of Edinburgh Institute (ZJU‐UoE Institute), International Campus Zhejiang University Haining China; ^5^ Edinburgh Medical School: Biomedical Sciences, College of Medicine and Veterinary Medicine The University of Edinburgh Edinburgh UK; ^6^ Biomedical and Health Translational Research Center of Zhejiang Province Haining China; ^7^ Department of Thoracic Oncology Hangzhou Cancer Hospital Hangzhou China; ^8^ Key Laboratory of Systems Biology, Shanghai Institute of Biochemistry and Cell Biology, Center For Excellence in Molecular Cell Science Chinese Academy of Sciences Shanghai China; ^9^ School of Electrical and Information Engineering Zhengzhou University Zhengzhou China; ^10^ State Key Laboratory of Intelligent Agricultural Power Equipment Zhengzhou University Luoyang China; ^11^ School of Mathematical Sciences and School of AI Shanghai Jiao Tong University Shanghai China

**Keywords:** causal inference, cell‐state transition, disentangled representation, drug resistance, network control

## Abstract

Understanding and controlling cell‐state and phenotype transitions is central to biological discovery and therapeutic development, yet identifying true causal regulators from observational transcriptomic data remains challenging because of confounding and correlated signals. CauFinder is a framework that integrates causal disentanglement modeling with network control to prioritize causal regulators of state and phenotype transitions from observed data. By leveraging causal reasoning based on do‐calculus and optimizing information‐flow metrics, CauFinder separates putative causal factors from spurious associations, quantifies transition‐relevant states, and nominates master regulators. Across simulations and multiple real‐world datasets, CauFinder identifies regulators associated with diverse transitions, including differentiation, adenocarcinoma‐to‐squamous transdifferentiation, and shifts between drug‐sensitive and drug‐resistant states. In epidermal growth factor receptor (EGFR) tyrosine kinase inhibitor (TKI) resistance, CauFinder prioritizes *DAAM1* as a previously unrecognized driver. Small interfering RNA (siRNA)–mediated knockdown of *DAAM1* enhances sensitivity to osimertinib, providing functional support for this causal prediction. Overall, CauFinder enables actionable target nomination and testable hypotheses for intervening in disease‐relevant state transitions using observational transcriptomic data.

## Graphical abstract

CauFinder combines causal disentanglement modeling and network control to prioritize causal drivers of cell‐state transitions from observational transcriptomic data. The framework separates transition‐relevant signals from spurious associations, nominates intervention targets across biological and disease contexts, and identifies *DAAM1* as an actionable driver in EGFR TKI resistance, whose knockdown enhances osimertinib sensitivity.

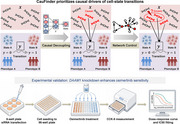

## 1 Introduction

Transitions in cell states and phenotypes, such as differentiation, reprogramming, or disease progression, are fundamental biological processes. Identifying the causal regulators underlying these transitions is critical for understanding disease mechanisms and designing effective interventions [1]. However, distinguishing causal drivers from correlated features remains a major challenge in observational transcriptomics, as association‐based methods can identify genes correlated with state transitions without establishing whether they causally drive the change.

Several computational approaches have been developed to infer regulatory influences from gene expression data, differing in assumptions, inputs and inferred relationships. One class of methods, gene regulatory network (GRN) inference, aims to reconstruct regulatory relationships between genes, such as GENIE3 [2] and PIDC [3]. Other approaches incorporate temporal information to model regulatory dynamics, including pseudotime‐based methods such as SCODE [4] and RNA velocity–based frameworks such as Velorama [5]. Cell conversion methods such as Mogrify [6] prioritize transcription factor combinations for predefined source‐to‐target reprogramming based on differential expression and regulatory network information. Another category focuses on identifying key regulatory drivers using network control principles, for example WMDS.net [7], which applies minimum dominating sets, and CEFCON [8], which integrates inferred regulatory networks with control theory to identify lineage‐specific regulators. Despite their utility, these approaches primarily rely on associations or inferred network topology rather than explicit causal modeling, limiting their ability to distinguish true causal drivers from correlated features.

Modern causal discovery approaches infer causal structure from observational data through different principles, including constraint‐based methods that use conditional independence relationships, such as the Peter–Clark (PC) algorithm [9] and FCI [10]; score‐based methods that search for graph structures by optimizing a scoring function, such as GES [11]; functional model–based methods that identify causal direction through assumptions on the functional relationship between causes and effects, such as LiNGAM [12]; and continuous‐optimization methods for structure learning, such as NOTEARS [13]. These methods have substantially advanced causal structure learning under different assumptions. However, they are primarily designed to infer causal relationships among variables, whereas transcriptomic state‐transition analysis requires identifying regulators that are specifically associated with transitions between biological states and evaluating their potential roles in phenotype modulation.

To address these challenges, we developed CauFinder, a deep learning‐based causal framework to prioritize candidate master regulators that exert global effects on cell‐state or phenotype transitions using observed data. CauFinder integrates causal modeling with network control to distinguish putative causal drivers from correlated features and to prioritize regulators with high intervention potential. We evaluate CauFinder in simulated, single‐cell, and bulk transcriptomic datasets across diverse biological transitions, including cell differentiation, lung adenocarcinoma (LUAD) to lung squamous cell carcinoma (LUSC) transdifferentiation, and drug‐sensitive to drug‐resistant transitions. We further demonstrate that CauFinder‐prioritized regulators can be experimentally validated, including the identification of *DAAM1* as a modulator of EGFR‐TKI response, where siRNA‐mediated knockdown significantly enhanced osimertinib sensitivity in pre‐treatment perturbation assays.

## 2 Results

### 2.1 Overview of CauFinder

**FIGURE 1:**
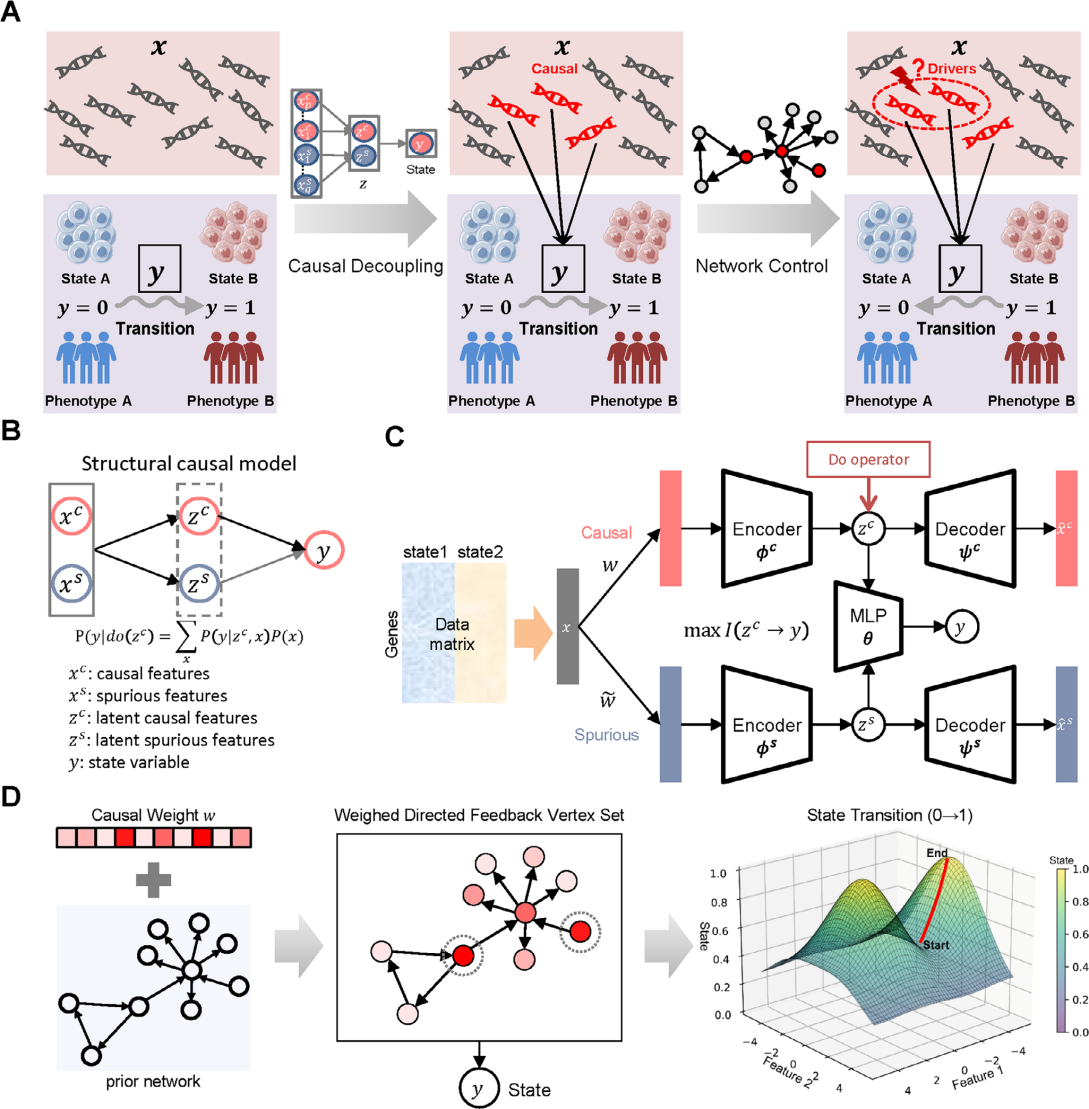
Overview of the CauFinder framework. (A) Schematic representation of the CauFinder framework for steering state or phenotype transitions by identifying key drivers through causal disentanglement followed by network control. (B) Structural causal model showing the decomposition of original features *x*  = {*x^c^*, *x^s^*} into their latent representations *z*  = {*z^c^*, *z^s^*} , with *y* denoting the state or phenotype. (C) Causal decoupling model constructed on a variational autoencoder (VAE) framework to identify causal drivers influencing state or phenotype transitions. Features *x* are processed through a feature selection layer, encoded to create a latent space *z*, segmented into causal (*z^c^*) and spurious (*z^s^*) components. This latent space is decoded to reconstruct the original features *x* as $\hat{x}$ and to predict the phenotype *y*. The model strives to maximize the causal information flow, $I(z^{c}\to y)$, from *z^c^* to *y*, thus delineating the causal pathways from *x* to *y* via *z^c^* and identifying the causal drivers for precise transition control. (D) Master regulator identification via causality‐weighted features and network control. Techniques including SHAP and gradient‐based methods are used to assign causality weights to features within the causal path defined in (C), aiding in the isolation of causal features for integration with prior network insights. Weighed directed feedback vertex set (FVS) is then employed to pinpoint master regulators critical for directing state or phenotype transitions through counterfactual generation for causal state transition, thereby establishing the foundation for targeted interventions.

The primary goal of CauFinder is to identify and control master regulators governing cell‐state or phenotype transitions. CauFinder elucidates state transitions by identifying causally relevant factors within a latent space and estimating causal information flow from latent features to state predictions, while accounting for confounders via backdoor adjustment [14]. Specifically, CauFinder comprises two key processes (Figure 1A): a causal disentanglement module and a network control module. The causal disentanglement module employs a variational autoencoder (VAE) architecture to separate causal and spurious elements in complex datasets. This model isolates features that directly influence cell‐state or phenotype transitions (Figure 1B and C), distinguishing causal relations from mere correlations. To connect latent causal signals to actionable targets, CauFinder employs SHapley Additive exPlanations (SHAP) [15] values and gradient calculations to identify key causal features in the input space and quantify their causal strength and direction on state transitions.

The network control module then integrates causal weights with prior network knowledge using a weighted directed feedback vertex set (FVS) method [16, 17] to prioritize candidate master regulators for these transitions (Figure 1D). CauFinder also supports counterfactual generation for exploring the feasibility of causal state transitions.

### 2.2 Benchmark Evaluation of CauFinder on Simulated Datasets

**FIGURE 2:**
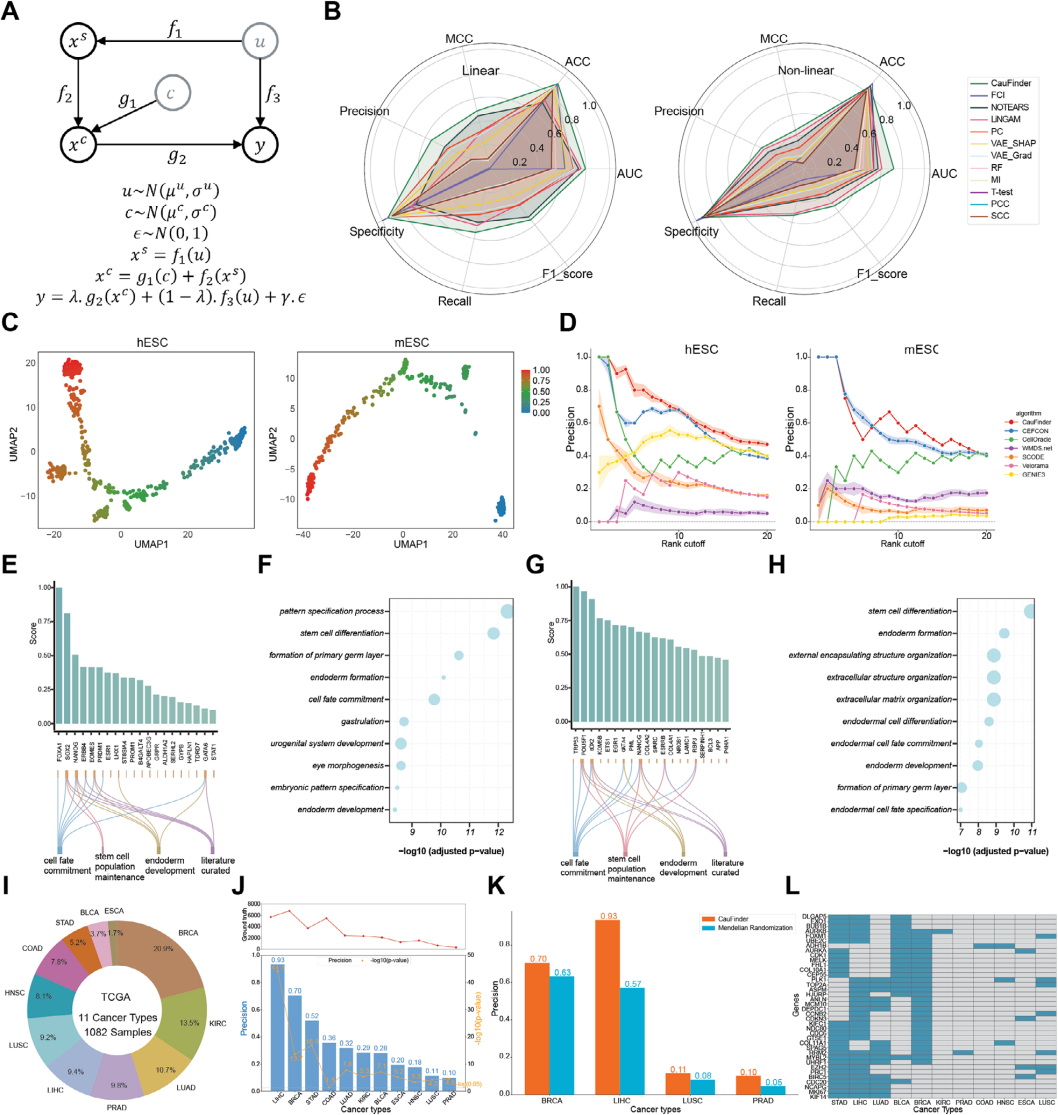
Evaluation of method performance in simulated and real‐world datasets. (A) Construction of synthetic simulation data. Here, *x^s^* represents the observed spurious variables, *x^c^* denotes the observed causal variables, and *y* is the outcome variable influenced by both *x^c^* and unobserved variables *u*. (B) Performance evaluation of CauFinder and comparative methods on simulated data in both linear (left) and nonlinear (right) scenarios. Radar charts show the average values of evaluation metrics (Accuracy (ACC), Matthews Correlation Coefficient (MCC), Area Under the Receiver Operating Characteristic Curve (AUC), Precision, Recall, Specificity, and F1‐score) for each method under different noise levels and causal strengths. (C) Pseudotime trajectories for hESC and mESC. The plots display the differentiation trajectories of human embryonic stem cells (hESC) and mouse embryonic stem cells (mESC) based on pseudotime analysis performed using Monocle 3. Cells are colored by pseudotime from early to late stages and visualized in a two‐dimensional embedding (UMAP). (D) Performance on the hESC and mESC datasets for each method, evaluated in terms of the precision of the top‐k predicted genes among all known genes in the ground‐truth gene set. The methods compared include CauFinder, CEFCON, CellOracle, WMDS.net, SCODE, Velorama and GENIE3. All the results with k ranking from 1 to 20 were reported. The shaded area represents the variation (mean ± s.d.) of precision over 10 repeats. (E–H) Identification and functional characterization of driver regulators in hESC and mESC differentiation. The top 20 predicted driver regulators in hESC (E) and mESC (G) are ranked in descending order according to their normalized causal weights inferred by CauFinder. Genes belonging to known ground‐truth regulator sets are indicated below the bar charts. Gene Ontology enrichment analysis of all predicted driver regulators in hESC (F) and mESC (H). Dot size represents the number of enriched driver genes, and terms are ranked by −log10(adjusted p‐value). *P* values were adjusted using the Benjamini–Hochberg method. (I) Donut chart showing the distribution of paired samples from different cancer types in the TCGA dataset analyzed. The size of each sector represents the proportion of samples for each cancer type. (J) Top red line chart representing the total number of pathogenic genes documented in the literature for each cancer type, sourced from the DisGeNET database. The bottom bars and line chart showing the precision (blue bars) of pathogenic genes identified by CauFinder in each cancer type, compared against literature‐documented pathogenic genes, and the ‐log10(p‐value) (orange line). The horizontal axis denotes different cancer types, the left vertical axis shows precision, and the right vertical axis shows the ‐log10(p‐value). (K) Bar chart comparing the precision of pathogenic gene identification by CauFinder and Mendelian Randomization (MR) in four cancer types: breast invasive carcinoma (BRCA), liver hepatocellular carcinoma (LIHC), lung squamous cell carcinoma (LUSC), and prostate adenocarcinoma (PRAD). Precision is defined as the proportion of predicted pathogenic genes that are supported by literature evidence. The orange bars represent the precision of CauFinder, and the blue bars represent the precision of Mendelian Randomization. (L) Heatmap illustrating the occurrence of genes identified by CauFinder as drivers in at least three different cancer types. The heatmap includes the top genes sorted by their frequency across 11 cancer types. The horizontal axis represents the cancer types, and the vertical axis lists the genes. Blue indicates that the gene was identified as a driver in a particular cancer type, while grey indicates that it was not identified as a driver in that cancer type.

Evaluating causal discovery is challenging because ground truth is rarely available. To enable controlled assessment, we constructed two simulated datasets: (i) a synthetic dataset with causal features *x^c^*, spurious features *x^s^*, and outcome *y*, with confounding and controlled causal strength/noise; and (ii) a perturbation‐based dataset generated by CausalRegNet [18] using real single‐cell expression to simulate unperturbed/perturbed profiles with phenotypes linked to perturbed genes (Figure 2A, Note S1). To evaluate causal feature identification, we compared CauFinder with representative baselines, including NOTEARS [13], FCI [10], LiNGAM [12], PC [9], random forest (RF), T‐test, variational autoencoder (VAE), mutual information (MI), Pearson correlation coefficient (PCC) and Spearman correlation coefficient (SCC). In these simulation‐based benchmarks, we evaluated only the causal disentanglement module of CauFinder, without incorporating prior network information or applying the downstream network control module. Since GRN inference is widely used in scRNA‐seq studies, we additionally included GENIE3, SCODE and Velorama in the perturbation‐based benchmark (Note S2). However, CauFinder fundamentally differs from GRN inference methods: it evaluates the influence of gene sets on state transitions, whereas GRN‐based methods were originally designed to infer pairwise regulatory interactions between genes rather than to prioritize regulators for state‐transition tasks. Therefore, these methods were included as cross‐paradigm references rather than strictly equivalent competitors (Figure S1, Table S1).

For the synthetic simulation data, we evaluated the performance of each method under different combinations of noise levels and causal strengths in both linear and nonlinear scenarios, with evaluation metrics and driver selection strategies detailed in Notes S3 and S4. CauFinder achieved the strongest overall performance across multiple evaluation metrics on average (Figure 2B). Several causal discovery methods, including NOTEARS, LiNGAM, and PC, also showed competitive performance, supporting the value of causal modeling in this benchmark. We further examined AUC values across different noise and causal‐strength combinations, which showed condition‐specific variability among methods (Figures S2, S3). As noise increased or causal strength decreased, the performance of all methods generally declined, while CauFinder maintained robust performance across different settings.

Furthermore, we analyzed CauFinder's causal decoupling behavior on the simulation dataset. In the latent space, the information flow measurement of the causal dimension was consistently higher than that of the spurious dimension, and causal features received higher weights than spurious ones (Figure S4A–C). While the original feature space showed limited class separation, the causal latent dimension yielded markedly clearer class differentiation than the spurious latent space (Figure S4D–E), demonstrating that CauFinder effectively disentangles causal and spurious factors and concentrates class‐discriminative information onto the causal dimension.

We further tested CauFinder on perturbation‐based simulation data, where gene perturbations directly influence the phenotype. Since GRN benchmarks (e.g. BEELINE [19]) emphasize network reconstruction rather than causal effects on state transitions, we evaluated GRN inference methods on the perturbation‐based dataset as cross‐paradigm references (Note S1). Under this perturbation‐driven setting, CauFinder achieved the strongest overall performance in both AUC and F1‐score among the compared methods (Figure S5), supporting its applicability to causal state transition inference. Additional analyses on hyperparameter sensitivity, ablations, and computational efficiency are reported in the Supplemental Information (Figure S6–S8; Note S5).

### 2.3 Benchmark Evaluation of CauFinder on Single‐cell and Bulk Datasets

To further validate CauFinder on real‐world data, we evaluated it on embryonic stem cell (ESC) single‐cell datasets and The Cancer Genome Atlas (TCGA) bulk datasets. We first evaluated CauFinder for driver regulator identification using mouse (mESC) [20] and human (hESC) [21] ESC datasets. As ground truth, we used CEFCON‐curated gene sets related to cell fate and ESC development [8], including cell fate commitment, stem cell population maintenance, endoderm development, and literature curated sets. Figure 2C shows the pseudotime trajectories of hESC and mESC, with cells colored from early to late stages of differentiation, inferred using Monocle [22]. Early and late stages were defined from normalized pseudotime, with threshold sensitivity summarized in Figure S9. We evaluated performance using top‐k precision and compared CauFinder with CEFCON, CellOracle [23], and WMDS.net (Note S2). In hESC, CauFinder achieved higher precision at early rank cutoffs, while CEFCON performed comparably at mid ranks; CellOracle and WMDS.net underperformed overall (Figure 2D). In mESC, CauFinder matched CEFCON at the earliest cutoffs and surpassed it thereafter, whereas WMDS.net consistently underperformed (Figure 2D).

We next examined the top 20 driver regulators identified by CauFinder in hESC and mESC, and nearly half overlapped with curated ground‐truth gene sets (Figure 2E and G). Specifically, the transcription factors (TFs) *SOX2* and *NANOG* ranked among the top drivers in both species and are established pluripotency regulators that form a cross‐regulatory feedforward loop [24, 25]. Additional pluripotency factors, including *EOMES* and *GATA6* in hESC and *POU5F1* and *GATA4* in mESC, also received high causal weights. Consistent with prior studies, *GATA4* and *GATA6* have been shown to promote primitive endoderm differentiation [26]. Beyond TFs, CauFinder also highlighted non‐TF drivers, including *ALDH1A2*, *PROM1*, *PML*, and *KDM5B*, implicated in ESC differentiation. For instance, *PML* has been reported to associate with a transcriptional repressive complex involving *OCT4* and *NANOG* and to contribute to ESC self‐renewal and pluripotency [27]. *ALDH1A2* is involved in retinoic acid (RA) synthesis and signaling [28]. *PROM1* is a marker of diverse stem and progenitor cells [29, 30, 31] and has been linked to responses to extracellular cues [32]. *KDM5B* is a KDM5‐family histone lysine demethylase [33] that has been reported to repress pluripotency genes, including *SOX2* and *NANOG* [34], and to act downstream of *NANOG* in regulating ESC pluripotency and self‐renewal [35].

We then performed Gene Ontology (biological process) enrichment analysis of the driver regulators identified by CauFinder. The top 10 pathways were selected and ranked by log‐transformed P‐values (Figure 2F and H). Several expected pathways were shared between species, including stem cell differentiation and endoderm formation, which were enriched in both mESC and hESC. Pattern specification processes were preferentially enriched in hESC (Figure 2F), whereas extracellular structure organization was enriched in mESC (Figure 2H). Together, these results indicate that CauFinder identifies transcription factors with strong species‐specific causal effects while also capturing downstream regulators and related enzymes implicated in ESC differentiation.

To further evaluate CauFinder on systems with known regulatory factors, we performed an additional benchmark using seven classical cell conversion settings previously examined in Mogrify [6], including fibroblast‐to‐neuron [36], fibroblast‐to‐myoblast [37], fibroblast‐to‐hepatocyte [38], fibroblast‐to‐iPSC [39], fibroblast‐to‐macrophage [40], fibroblast‐to‐cardiomyocyte [41], and B cell‐to‐macrophage transitions [42] (Figure S10). These conversion systems and their associated regulatory factors were curated from the Mogrify benchmark and traced back to the original experimental studies. For each method, we examined the recovery and ranking of these literature‐supported regulators within the predicted candidate gene lists. CauFinder recovered multiple established regulators and showed competitive performance compared with existing baseline methods. For Mogrify, whose commercial implementation is not publicly reproducible, we used the regulator lists and reported conversion examples from the original publication for comparison [6].

Following these analyses, we evaluated CauFinder on The Cancer Genome Atlas (TCGA) bulk data across multiple cancer types (Figure 2I) to identify putative cancer drivers. We analyzed paired tumor and adjacent normal samples from 11 TCGA cancer types [43] (over 1000 samples in total). For validation, we utilized a set of ground truth data sourced from the DisGeNET [44] database, which provides a comprehensive collection of genes and variants associated with human diseases. We assessed accuracy by the overlap between CauFinder‐predicted drivers and DisGeNET pathogenic genes. CauFinder achieved high precision in recovering DisGeNET‐supported cancer drivers (Figure 2J). Notably, precision increased with the number of available ground‐truth genes, consistent with incomplete annotations in some cancer types and suggesting that CauFinder may prioritize plausible drivers even when curated ground truth is sparse.

To further benchmark CauFinder, we compared it with Mendelian randomization (MR) [45], which uses genetic variants as instrumental variables for causal inference. We focused on four cancer types with available single‐nucleotide polymorphism (SNP) data. Across all four cancer types, CauFinder achieved higher precision than MR (Figure 2K), indicating improved recovery of literature‐supported drivers in this setting. Notably, CauFinder does not require SNP information, enabling application when genetic instruments are unavailable. We also examined drivers recurrently identified across cancer types (Figure 2L). Recurrently identified genes included *PLK1* and *RRM2*, which have been widely implicated in tumorigenesis and cancer progression [46, 47, 48, 49, 50, 51, 52]. This supports CauFinder's ability to recover known drivers and to highlight recurrent candidates shared across cancer types.

### 2.4 Inferring Candidate Regulators of Lung Adeno‐To‐Squamous Transdifferentiation

Human lung adenosquamous carcinoma (LUAS) is a distinct NSCLC subtype reported to account for ≈0.7%–11.4% of cases and is associated with high plasticity, therapy resistance, and poor prognosis compared with lung adenocarcinoma (LUAD) and lung squamous cell carcinoma (LUSC) [53, 54, 55, 56, 57, 58, 59, 60]. A recent study analyzing the largest LUAS cohort to date suggested that LUAS can arise via transdifferentiation from LUAD toward a squamous phenotype [61]. In that dataset, samples were stratified by marker genes into three subtypes: TRU‐like (LUAD‐like), inflammatory, and basal‐like (LUSC‐like). Using this dataset, we asked whether CauFinder could identify candidate regulators associated with adeno‐to‐squamous transdifferentiation and generate in silico hypotheses regarding transition‐associated regulatory programs.

**FIGURE 3:**
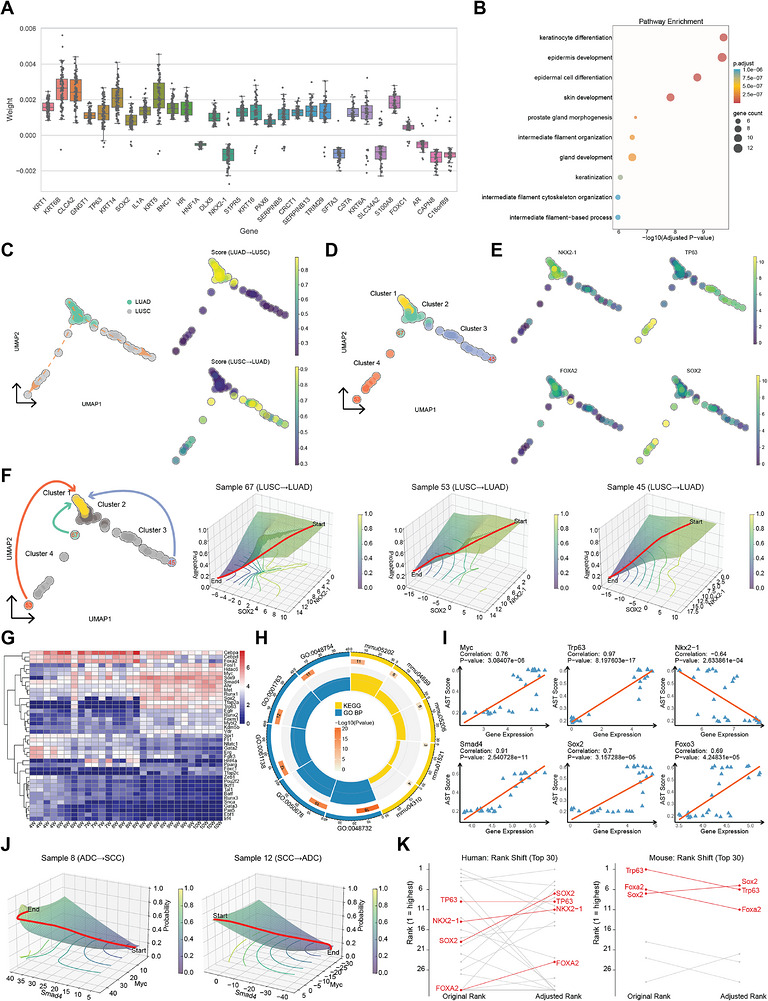
Identification of candidate regulators associated with lung adeno‐to‐squamous transdifferentiation (AST). (A) Boxplots with individual data points showing the distribution of CauFinder causal weights for the top 30 genes in the human LUAS cohort, ranked by frequency of occurrence across 100 runs. (B) Pathway enrichment analysis of the top 30 genes identified in (A). Bubble size represents gene count, and bubble color represents adjusted *p* value. (C) Uniform manifold approximation and projection (UMAP) of human LUAS RNA‐seq samples, colored by tumor subtype (left) or by CauFinder path controllability score for the forward (LUAD→LUSC; top right) and reverse (LUSC→LUAD; bottom right) directions. (D) UMAP plot showing the clustering results of samples using the Leiden algorithm with a resolution of 0.25. (E) The expression patterns of four previously reported regulators identified by CauFinder are shown across the samples on the same UMAP coordinates as in (D). These genes exhibit distinct expression profiles between LUAD and LUSC, as well as along two trajectories within LUSC. (F) In silico counterfactual simulations of selected samples under control of causal genes. The leftmost panel shows three LUSC‐like samples selected for transition toward LUAD, and the remaining panels show the corresponding trajectories for samples 67, 53, and 45. Each panel illustrates changes in predicted state probability during paired gene‐expression perturbations, where 0 indicates proximity to LUAD and 1 indicates proximity to LUSC. (G) Heatmap of driver gene expression during lung adeno‐to‐squamous transdifferentiation (AST) in the mouse model. The horizontal axis shows weeks post Ad‐Cre nasal inhalation (4, 6, 7, 8, 9, and 10 W), and the vertical axis shows driver genes identified by CauFinder. (H) Circos plot of KEGG and GO enrichment analysis for AST‐related genes. The plot shows the top 10 enriched pathways, with sector size proportional to gene count. Yellow sectors indicate KEGG pathways and blue sectors indicate GO pathways. From outer to inner rings, the plot shows pathway ID, gene count colored by ‐log10 *p* value, and GeneRatio (the ratio of enriched genes to total input genes). Wnt signaling pathway (mmu04310) is highlighted. (I) Scatter plots showing Pearson correlation between gene expression and path controllability scores of each AST sample. The horizontal axis shows gene expression levels, and the vertical axis displays samples path controllability scores provided by CauFinder. (J) In silico counterfactual simulations of state transitions (between LUAD and LUSC) via control of Myc and Smad4. The left panel shows the predicted transition from LUAD to LUSC in a 7 W sample, and the right panel shows the predicted transition from LUSC to LUAD in a 9 W sample. (K) Rank‐shift plots comparing CauFinder gene rankings before and after tumor purity and microenvironmental adjustment in the human LUAS cohort (left) and the mouse AST model (right). Key regulators, including *TP63, SOX2, NKX2‐1*, and *FOXA2*, remained among the top‐ranked candidates after adjustment.

We defined LUAD‐like LUAS samples as the starting state (state 0) and LUSC‐like samples as the endpoint (state 1). CauFinder then prioritized candidate regulators of adeno‐to‐squamous transdifferentiation (AST) (Figure 3A; Figure S11A, B), including previously validated factors such as *TP63*, *SOX2*, *NKX2‐1*, and *FOXA2* [61]. Pathway enrichment further supported the biological relevance of the prioritized candidates (Figure 3B). We compared CauFinder with WMDS, CEFCON, and CellOracle. CauFinder and CEFCON showed the highest overlap (Figure S12A) and yielded consistent enrichment for development‐ and morphogenesis‐related processes (Figure 3B; Figure S12B), suggesting convergence on core programs associated with squamous epithelial transdifferentiation. Notably, CauFinder additionally highlighted epidermal development, differentiation, and keratinization terms and prioritized canonical squamous markers such as *KRT5* and *KRT14* [62, 63]. To characterize sample structure, we performed UMAP visualization and clustering (Figure 3C and D). The resulting embedding revealed an inferred LUAD‐to‐LUSC continuum with two LUSC‐branching trajectories that converge on a squamous‐like endpoint, consistent with previous findings [61].

We inspected path controllability scores for both the forward (LUAD→LUSC) and reverse (LUSC→LUAD) directions across LUAS samples (Figure 3C). LUAD samples showed uniformly high scores for LUAD→LUSC, whereas LUSC samples showed high scores for LUSC→LUAD only along one trajectory, suggesting heterogeneous predicted reversal potential. Clustering further separated LUAD and LUSC samples (Figure 3D; Figure S11C, D), with one LUSC sample grouping with LUAD and showing the highest score. Across clusters, both LUAD and LUSC contained a subcluster with significantly higher transition scores (Figure S11E), indicating a subset of samples with elevated transition potential, consistent with previous observations [61]. Driver‐gene expression broadly tracked path controllability, with gene‐specific variation (Figure 3E). We then performed in silico counterfactual modulation by adjusting two marker genes, NKX2‐1 and SOX2, to shift the predicted state probability toward LUAD. Across three LUSC samples, this in silico modulation increased the predicted LUAD‐like probability (Figure 3F), with samples 67 and 45 showing larger changes than sample 53, consistent with their higher path controllability scores.

We next investigated candidate mechanisms associated with AST. Prior work implicates Wnt signaling in maintaining the LUAD–LUSC lineage balance and shows that Wnt inhibition promotes adeno‐to‐squamous transdifferentiation, but the regulatory mechanisms remain unclear [64]. We therefore applied CauFinder to a published Ad‐Cre–induced mouse AST model [64], defining 4–7 weeks post induction (4 W, 6 W, 7 W) as LUAD stages, 8 W as an intermediate stage, and 9–10 W (9 W, 10 W) as LUSC stages. Within this framework, we defined LUAD stages as the start state (state 0) and LUSC stages as the end state (state 1), and also evaluated the reverse transition (LUSC→LUAD), holding out the intermediate stage (8 W) to assess generalization along the transition. CauFinder prioritized candidate regulators associated with AST (Figure 3G), including previously validated factors such as *Foxa2*, *Sox2*, and *Trp63* [61, 64]. Gene Ontology (biological process) enrichment highlighted gland development (GO:0048732), regulation of epithelial cell proliferation (GO:0050678), and branching morphogenesis (GO:0061138, GO:0001763, GO:0048754) among the top terms (Figure 3H). KEGG enrichment also identified Wnt signaling (mmu04310), consistent with prior reports (Figure 3H).

We next mapped candidate genes to the significantly enriched pathways to pinpoint potential mechanisms. Previously validated regulators (*Trp63*, *Sox2*, *Foxa2*) overlapped with pathways related to gland development, regulation of epithelial cell proliferation, and branching morphogenesis. Notably, Wnt‐pathway genes also intersected these processes: *Myc* mapped to regulation of epithelial cell proliferation and branching morphogenesis, *Smad4* to branching morphogenesis and gland development, and *Nfatc1* to regulation of epithelial cell proliferation and branching morphogenesis. Although *Nkx2‐1* was not prioritized in this setting by CauFinder, the identification of *Smad4* as an upstream regulator with inhibitory effects [65] suggests that *Nkx2‐1* may be involved downstream or in parallel within this regulatory context.

We performed PCA on LUAD and LUSC samples across time points to assess global transition structure. Samples were ordered by progression stage along the principal component space (Figure S11F). Samples near the previously reported DNB‐defined tipping point [64] showed elevated path controllability scores (Figure S11G and H), consistent with increased transition susceptibility. Expression of *Myc* and *Smad4* was associated with AST path controllability scores (Figure 3I). In addition, in silico counterfactual modulation of *Smad4* and *Myc* altered the predicted transition scores in selected samples (Figure 3J; Figure S13), suggesting potential shifts in AST propensity within the computational model. These results are observationally derived counterfactual predictions and do not establish biological necessity or sufficiency. Experimental perturbation in appropriate LUAD/LUSC models will be required to test these predictions.

To further assess the robustness of AST‐related signals against bulk compositional confounding, we estimated tumor purity and microenvironmental composition in both the human LUAS cohort and the mouse AST model using ESTIMATE [66] and xCell [67]. We then regressed out tumor purity, stromal/immune scores, and major microenvironmental components and reran CauFinder on the adjusted expression matrices. The key prioritized regulators remained highly ranked after adjustment, despite substantial reshuffling of many background genes (Figure 3K; Figure S14), supporting robustness to tumor purity and inferred stromal/immune variation. The reduced correlations between representative core regulators and tumor purity after adjustment further suggest that their prioritization is unlikely to be driven solely by compositional variation.

### 2.5 CauFinder Identifies Regulatory Mechanisms of Lung Cancer Cycling Persister Cells and Guides Improvement of Drug Response

To further assess the robustness and temporal versatility of CauFinder, we used a multi‐time‐point dataset from a previous study [68] that performed lineage tracing in PC9 lung cancer cells, with a focus on cycling persister cells.

**FIGURE 4:**
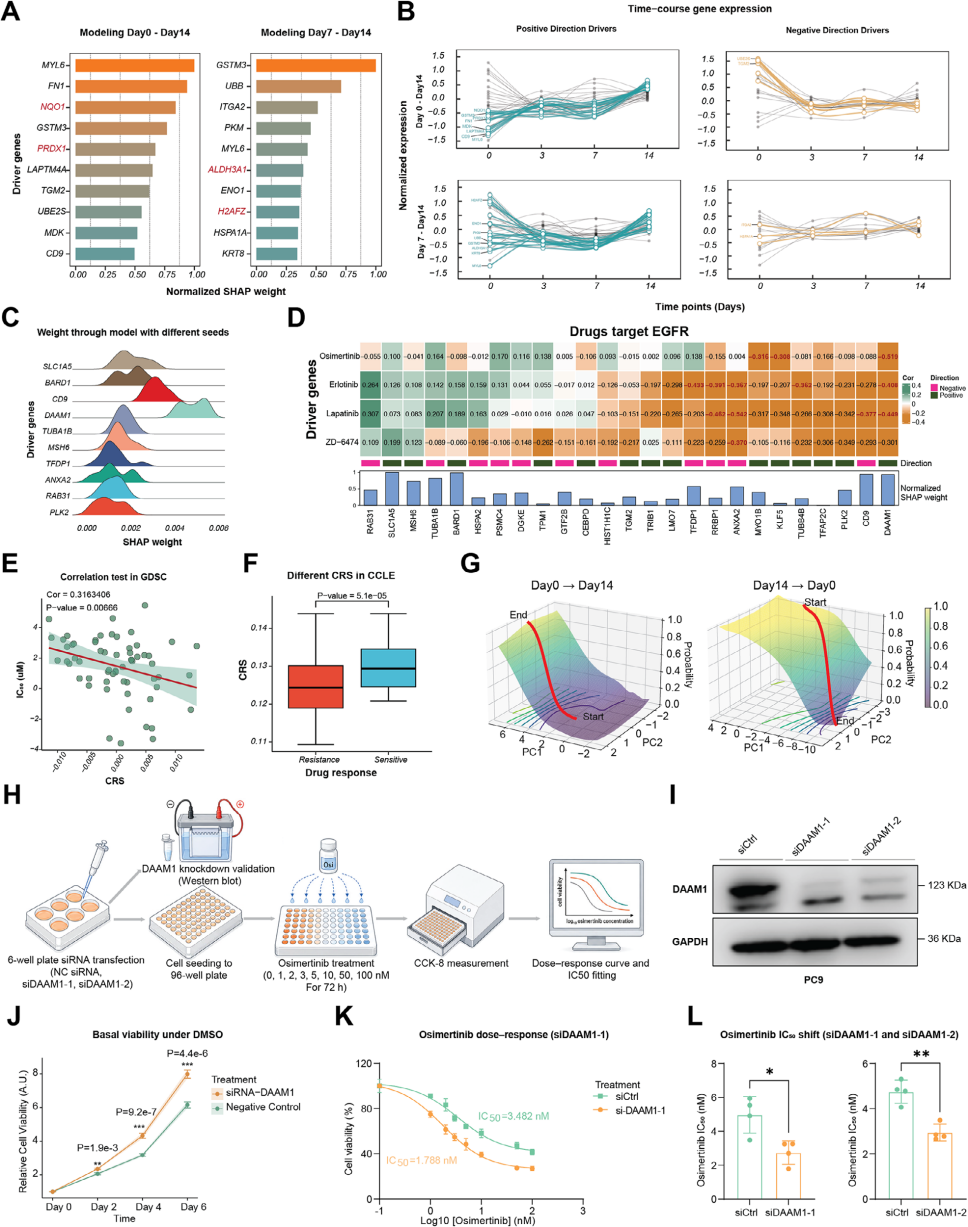
Characterizing causal gene expression programs associated with cycling persister cells emergence and responses to EGFR‐targeting drugs. (A) Top ten causal drivers identified by CauFinder, based on lineage barcode tracing of progenitor cells of cycling persister cells sequenced on day 0 (left) and day 7 (right), paired with corresponding cycling persister cells on day 14 as the input. The drivers are ranked by their normalized SHAP weights. Genes involved in the ROS and FAM pathways are highlighted in red. (B) Time‐course expression of causal driver genes identified by CauFinder for two different paired inputs. Expression is represented as the normalized average expression of the corresponding genes across all lung cancer cells in each time category. Causal drivers are grouped by the direction of their weights from the CauFinder output, with the top ten drivers highlighted. Drivers with positive weights are shown in blue, while those with negative weights are shown in orange (Results from day 0 to day 14 input, upper; results from day 7 to day 14 input, lower). (C) Top ten causal drivers identified by CauFinder, based on results from using day‐0 sensitive‐lineage ancestors paired with day‐0 resistant‐lineage ancestors as input. The density estimation of SHAP weights output by CauFinder is shown for each causal driver, with different seeds used for robustness. The drivers are ranked by their SHAP weights. (D) Heatmap showing the Pearson correlation between drug response IC_50_ values of cell lines tested with four EGFR‐targeting drugs in the GDSC and CCLE databases and the expression of each causal driver gene across cell lines (Osimertinib from GDSC; other EGFR inhibitors from CCLE). Correlation pairs with a *p* value less than 0.05 are highlighted. The color bar indicates the direction of the causal drivers' weights as determined by CauFinder. The bar plot at the bottom represents the normalized SHAP weights of the corresponding causal drivers. (E) Correlation between causal drug response score (CRS) and IC_50_ values (horizontal and vertical axes) in cell lines (dots) from the GDSC database. (F) Boxplots showing the distribution of CRS of cell lines profiled for three EGFR‐targeting drugs in the CCLE database across two drug response groups. *P* values were obtained from the t‐test. The middle horizontal line represents the median value. Each box spans the lower quartile to the upper quartile. The whiskers indicate the minimum and maximum values within 1.5 times the interquartile range (IQR). (G) Computational simulation of state transitions for different samples from the time‐course pairs. Principal components (PCs) identified through principal component analysis (PCA) of all drivers. The X‐axis and Y‐axis represent the principal components (PCs) of these controlling drivers, while the Z‐axis indicates the state probability, where values closer to 0 represent the defined starting point and values closer to 1 represent the defined endpoint. (H) Experimental workflow for evaluating the role of *DAAM1* in EGFR‐TKI response. PC9 cells were transfected with control (siCtrl) or *DAAM1*‐targeting siRNAs (siDAAM1‐1 and siDAAM1‐2), followed by knockdown validation, drug treatment, viability measurement, and IC_50_ estimation. (I) Western blot validation of *DAAM1* knockdown in PC9 cells. GAPDH serves as a loading control. (J) Basal cell viability of PC9 cells under DMSO treatment following transfection with control or *DAAM1*‐targeting siRNAs, indicating no reduction in viability upon *DAAM1* knockdown. Data are presented as mean ± SD (n = 3 independent experiments). (K) Representative osimertinib dose–response curve in PC9 cells following siDAAM1‐1 knockdown compared to control cells. (L) Quantification of osimertinib IC_50_ values in PC9 cells following knockdown with two independent siRNAs (siDAAM1‐1 and siDAAM1‐2), demonstrating consistent IC_50_ reduction. Data are presented as mean ± SD from independent experiments, and statistical significance was assessed using unpaired two‐sided t‐tests (*p < 0.05, **p < 0.01).

We constructed four biologically grounded paired‐state settings to infer causal drivers (including two lineage‐based contrasts and two cycling versus non‐cycling contrasts; Note S6). Across these inputs, CauFinder consistently identified drivers enriched in reactive oxygen species (ROS) and fatty acid metabolism (FAM) pathways (Figure 4A; Figure S15). Across paired inputs with comparable biological context, the inferred drivers showed consistent enrichment in these biological processes (Figure S15B). This consistency across random seeds supports the robustness of CauFinder (Figure S15A). Consistent with these results, temporal expression analysis of drivers from the two lineage‐based inputs (day0 to day14 and day7 to day14) followed the direction of their inferred weights (Figure 4B): positively weighted drivers increased toward the target state (day14), whereas negatively weighted drivers showed the opposite trend, indicating directionally consistent regulation over time.

We further partitioned the same dataset by tumor cell drug resistance using lineage tracking (Note S6). CauFinder prioritized resistance‐associated drivers, including *CD9*, *BARD1*, and *SLC1A5* (Figure 4C; Table S2). We explored the correlation between the expression of all candidate causal drivers and the IC_50_ of four EGFR‐targeting drugs in the CCLE [69] and GDSC [70] datasets (Figure 4D). Significant correlations were observed for several genes, including *TFDP1, RRBP1, ANXA2, DAAM1*, with IC_50_ values for one or more drugs (Pearson correlation, *p* value < 0.05). This indicates that CauFinder can capture a variety of genes related to drug sensitivity. However, not all drivers showed significant single‐gene correlations with IC_50_, suggesting that these drivers may act collectively rather than independently.

We constructed a causal drug response score (CRS) by weighting gene expression with CauFinder‐inferred driver weights and aggregating signals using the inferred sign of each driver. CRS was significantly associated with IC_50_ values for EGFR‐targeting drugs in the GDSC and CCLE datasets and effectively stratified cell lines by drug sensitivity (Figure 4E, F and Figure S16G, H; Note S6). To directly assess its relevance to acquired drug resistance, we applied CRS to independent paired TKI‐sensitive and TKI‐resistant cell‐line datasets [71, 72], where CRS was consistently elevated in resistant cells (Figure S17A, B). In TCGA lung cancer cohorts, CRS showed supportive associations with overall survival but should be interpreted as reflecting prognostic tumor biology rather than treatment‐specific predictive utility because treatment exposures were heterogeneous and largely undocumented (Figure S17C–F).

CauFinder's capability to explore sample state transitions can also be applied to single‐cell data. To address the sparsity and large cell numbers, we summarized each state by pseudo‐cells obtained via within‐state reclustering and mean expression, which reduces computational burden while retaining dominant state‐level signals. We transformed the three time‐point data (day0, day7, and day14) into pseudo‐cells (Figure S16C and I; Note S7), and generated pseudo‐cells for drug‐sensitive and resistant states (Figure S16D and J; Note S7) to capture state‐level features.

To demonstrate CauFinder's capability in modeling complex state transitions, we evaluated bidirectional trajectories: forward (targeting day 14) and reverse (initiating from day 14). Using the path controllability score, we found that lineage‐intermediate states (day 7) exhibited significantly higher plasticity than initial states (day 0) in both directions (Figure S16L, M; *p* < 0.05). Simulations further highlighted the theoretical potential for guiding reverse transitions from the persister state (Figure 4G). Notably, CauFinder identified regulatory heterogeneity within the initial population; for instance, pseudo‐cell 4 in day 0 displayed elevated controllability scores (0.045) linked to the differential expression of *POLR3A* and *COX19* (Figure S16E, F), suggesting specific early markers of transition potential.

Next, we extended this modeling to drug response phenotypes by generating pseudo‐cells for sensitive and resistant states. By manipulating high‐confidence drivers identified by CauFinder, specifically *DAAM1* and *SLC1A5*, we efficiently simulated state transitions that altered drug sensitivity (Figure S18A, B). Crucially, comparison of these bidirectional paths revealed a kinetic asymmetry: while forward acquisition of drug resistance followed relatively straightforward regulatory trajectories, the simulated reverse shift toward sensitivity involved substantially more complex and difficult‐to‐regulate paths (Figure S16K). This computational insight aligns with the clinical observation that resistance is more readily acquired, whereas restoring sensitivity requires overcoming substantial biological barriers.

CauFinder prioritized *DAAM1* as one of the candidate regulators of osimertinib response, with consistently high SHAP weight across models initialized with different random seeds (Figure 4C). *DAAM1* was therefore selected for experimental validation in PC9 cells. To reduce the possibility that the observed phenotype was driven by a single siRNA‐specific off‐target effect, PC9 cells were transfected with negative control siRNA or two independent *DAAM1*‐targeting siRNAs, followed by synchronized Western blot validation and osimertinib dose‐response assays (Figure 4H). Western blotting confirmed that both *DAAM1*‐targeting siRNAs reduced DAAM1 protein expression compared with the negative control, with GAPDH as the loading control (Figure 4I). *DAAM1* knockdown did not reduce basal viability under DMSO treatment, arguing against nonspecific growth suppression as the basis for the drug‐response phenotype (Figure 4J). Full osimertinib dose‐response analysis showed a leftward shift of the response curve following *DAAM1* knockdown (Figure 4K). This effect was reproduced across four independent biological replicates for each *DAAM1*‐targeting siRNA (Figure S19), and comparison of fitted IC_50_ values confirmed a significant reduction in osimertinib IC_50_ after DAAM1 knockdown (Figure 4L). Together, these results indicate that *DAAM1* knockdown enhances osimertinib sensitivity in parental PC9 cells.

Taken together, these experimental findings directly corroborate that *DAAM1*, identified by CauFinder as a candidate causal driver, plays a functional and context‐dependent role in modulating cellular sensitivity to osimertinib, thereby validating the biological relevance of this CauFinder‐prioritized regulator. Building on this proof‐of‐concept, CauFinder‐derived driver sets can help nominate potential gene targets whose modulation may enhance drug sensitivity and have the potential to inform future therapeutic strategies for treating drug‐resistant cancers.

### 2.6 CauFinder Defines Key Intermediate Cell States and Candidate Interaction Mechanisms in Primary Colorectal Cancer (P1) and Paired Liver Metastasis (LM1)

**FIGURE 5:**
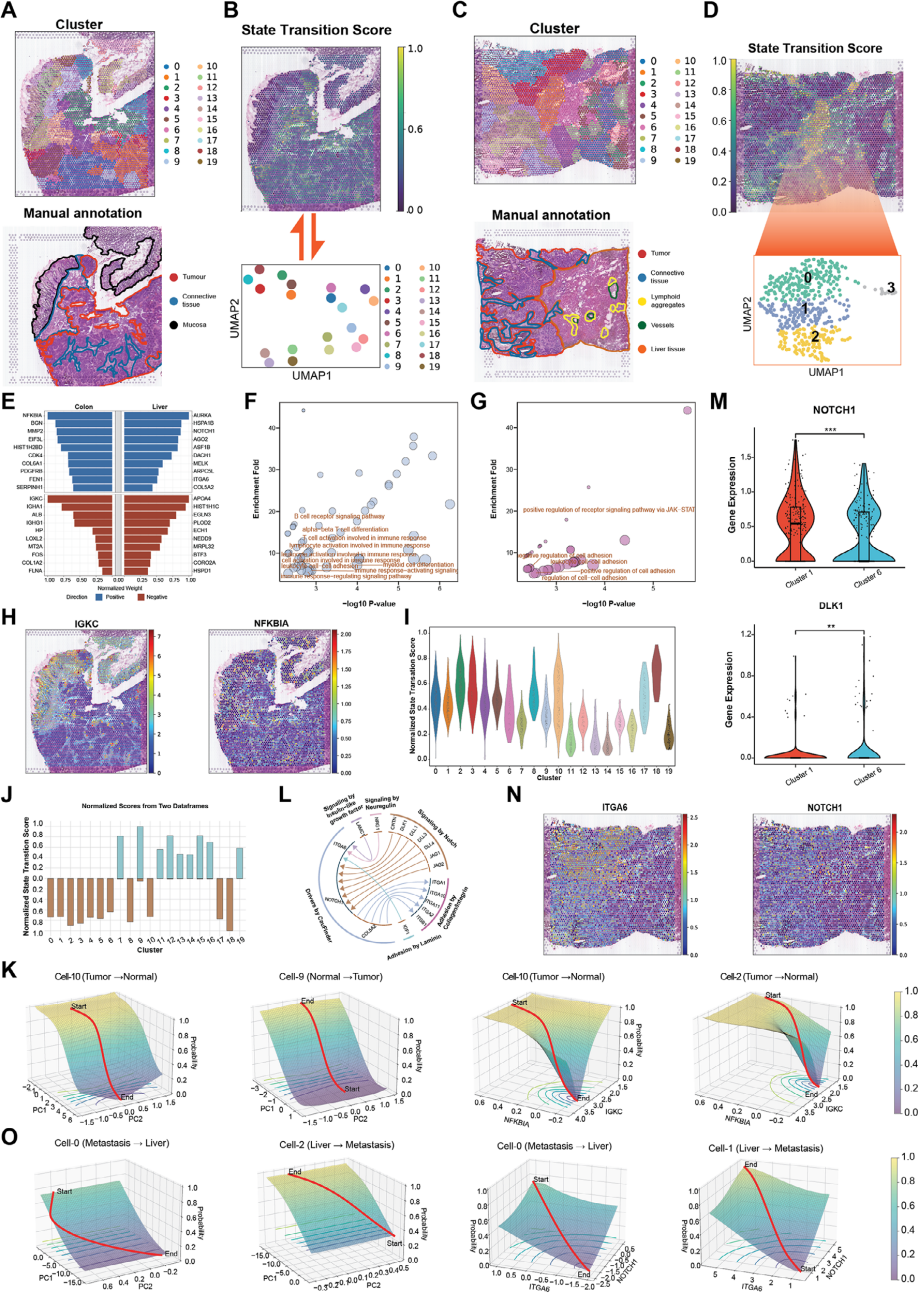
Identification of candidate driver genes and predicted intercellular interactions in colorectal cancer spatial transcriptomics. (A) Hematoxylin and eosin (H&E) plot of P1 tissue (top) with manual annotation of three regions: tumor (red), connective tissue (blue), and mucosa (black). Spatial clustering by MCGAE on P1 tissue (bottom). (B) Spatial distribution plot of cell path controllability scores of P1 tissue (top). Cells are re‐clustered and converted to pseudo‐cells, which are then used as input for CauFinder‐based state‐transition simulation (bottom). (C) Hematoxylin and eosin (H&E) plot of LM1 tissue (top) with manual annotation of five regions: tumor (red), connective tissue (blue), lymphoid aggregates (yellow), vessels (green), and liver tissue (orange). Spatial clustering by MCGAE on LM1 tissue (bottom). (D) Spatial distribution plot of cell path controllability scores of LM1 tissue (top). Cells are re‐clustered and converted to pseudo‐cells, which are then used as input for CauFinder‐based state‐transition simulation (bottom). (E) Top ten causal drivers with positive and negative weights defined by CauFinder in P1 tissue (left) and LM1 tissue (right). Bar length indicates the absolute value of the weights, with color representing the direction: positive (blue) and negative (red). (F) Bubble plot of GO BP enrichment analysis using all identified causal driver genes in P1 tissue, with immune‐related pathways labeled. The size of each point represents the number of genes enriched in the pathway, the horizontal axis shows the log‐transformed p‐value, and the vertical axis indicates the enrichment fold (enrichment fold = GeneRatio/BgRatio, provided by clusterProfiler). (G) Bubble plot of GO BP enrichment analysis using all identified causal driver genes in LM1 tissue, presented in the same manner as in (F). (H) Spatial expression of *IGKC* and *NFKBIA* genes in P1 tissue. (I) Violin plot showing cell path controllability scores among different clusters, determined by clustering results, with each cluster represented by a different color. (J) Cell path controllability scores of pseudo‐cells, converted from single cells by cluster, simulating transition to two states (tumor: 1, normal tissue: 0). Blue indicates transition to the tumor state and red indicates transition to the normal tissue state. (K) Computational simulation of state transitions in a subset of pseudo‐cells from P1 tissue. Principal components (PCs) identified through principal component analysis (PCA) of all drivers. The X‐axis and Y‐axis represent the principal components (PCs) of these controlling drivers, while the Z‐axis indicates the state probability, where values closer to 0 represent the defined starting point and values closer to 1 represent the defined endpoint. (L) Circos plot of predicted ligand‐receptor interactions involving causal drivers in LM1 tissue, with each arrow pointing from ligand to receptor. The outermost text represents different ligand‐receptor sets as described in CellPhoneDB. (M) Expression of NOTCH1 gene and its ligand DLK1 at the boundary between two clusters of tumor and normal tissue. (N) Spatial expression of *NOTCH1* and *ITGA6* in LM1 tissue. (O) Computational simulation of state transitions in a subset of pseudo‐cells at the tumor‐liver tissue interface in LM1 tissue. The X‐axis and Y‐axis represent the same variables as in (K).

To evaluate the potential of CauFinder in spatial transcriptomics analysis, we selected a dataset of primary colorectal cancer (P1) and paired liver metastasis (LM1) from a recent study [73] (Figure 5A–C). For the primary colorectal cancer sample P1, we initially applied the MCGAE [74] algorithm to cluster the spatial transcriptomics data. We retained only the tumor tissue and normal tissue sections as input for training the CauFinder model (Figure 5A and Note S6). For LM1, we selected two specific cell clusters at the tumor‐liver interface (Cluster 1 and Cluster 6) to represent tumor and liver tissue inputs, respectively (Figure 5C and Note S6).

We trained models using the selected tumor and normal inputs and identified high‐confidence causal drivers that appeared consistently across ten random seeds, supporting algorithmic robustness (Figure 5E). The results revealed distinct regulatory landscapes: P1 drivers were enriched in immune‐related pathways (identifying genes such as *IGKC* and *NFKBIA*), whereas LM1 drivers were enriched in ligand–receptor and adhesion‐related mechanisms (identifying *NOTCH1* and *COL5A2*) (Figure 5F and L). Moreover, mapping LM1 drivers to the CellPhoneDB [75] cell communication database indicated multiple interaction pairs. Among these, the *DLK1* and *NOTCH1* axis showed opposite differential expression between adjacent tumor and liver tissues, suggesting potential reciprocal regulation (Figure 5M, N). These findings suggest that CauFinder can characterize immune‐associated transitions in primary colorectal cancer (P1) and cell–cell communication drivers in liver metastasis (LM1). We independently simulated state transitions for all single cells using the identified causal drivers, determining the predicted transition direction based on the higher path controllability score (Figure 5B–D).

In the P1 sample, path controllability scores were spatially concentrated in the connective tissue region between the tumor and mucosa, and in adjacent tumor cells. This spatial distribution aligned with the expression of key drivers: *IGKC* was enriched in mucosa/connective tissue, while *NFKBIA* was highly expressed in tumor tissue (Figure 5H and I). To dissect these transitions, we converted cells into pseudo‐cells (Figure 5B; Note S7). Simulations revealed distinct fates: connective tissue‐derived pseudo‐cells (e.g., cell‐9) exhibited strong tumor transition potential, whereas tumor‐associated pseudo‐cells (e.g., cell‐10) could be regulated toward a normal state but not a tumor state (Figure 5K; Figure S20A, B). By modulating high‐SHAP‐value drivers (*IGKC*, *NFKBIA*), we simulated predicted state shifts in boundary regions (Figure S20C), demonstrating CauFinder's ability to nominate spatial transition‐associated factors and generate candidate targets for future therapeutic investigation.

In the LM1 sample, high path controllability scores were concentrated at the tumor‐liver interface (Cluster 1) (Figure 5D). Crucially, these cells were not labeled during model training, indicating that CauFinder identified transitional states without supervision. Simulations on interface pseudo‐cells were consistent with their metastatic origin, showing strong tumor maintenance potential with no transition to liver‐like states (Figure S20D). Furthermore, in silico modulation of specific drivers, such as *ITGA6* and *NOTCH1*, predicted that interface cells (e.g., cell 0 and cell 1) could shift toward a normal state (Figure 5O and Figure S20E). The probability differences in these simulated transitions reflect the balance between promoting and inhibiting invasion, suggesting that early‐stage boundary tumors may exhibit an inhibitory tendency that could be explored through future gene‐targeted perturbation studies.

## 3 Discussion

Understanding and manipulating cell states and phenotype transitions is central to both basic biology and therapeutic development. Here we introduce CauFinder, a framework that identifies candidate causal regulators of cell‐state/phenotype transitions and evaluates transition feasibility by integrating causal disentanglement with nonlinear network control using observational data. Conceptually, CauFinder prioritizes a minimal regulator set for a specified transition and assesses whether targeted modulation could plausibly induce the desired state change within the computational model.

Across simulated and real‐world datasets, CauFinder identified causal features/genes associated with phenotype transitions, quantified transition feasibility, and supported in silico exploration of potential control and reversal strategies. When available, incorporating prior interaction knowledge further improved robustness. We also quantify transition feasibility using a path controllability score. Notably, CauFinder captured natural biological transitions (e.g., differentiation, LUAD‐to‐LUSC transdifferentiation, and drug‐sensitive‐to‐resistant processes) and highlighted regulators associated with reverse tendencies, including experimentally supported enhancement of osimertinib sensitivity following *DAAM1* knockdown.

CauFinder is conceptually distinct from methods that either reconstruct gene–gene interactions or simulate perturbation outcomes. GRN inference approaches (e.g., GENIE3, SCODE, and Velorama) estimate transcription factor–target relationships from statistical dependencies, but do not explicitly evaluate whether modifying a factor is necessary and sufficient to drive a phenotypic transition. Counterfactual perturbation simulators such as CellOracle instead ask “What happens if I perturb TF *X*?” by predicting downstream consequences of a specified perturbation. In contrast, CauFinder prioritizes a minimal set of candidate regulators required for a target transition (“Which variables are needed to move from state 1 to state 2?”) and evaluates whether the transition is feasible via network control theory, thereby complementing perturbation‐based simulators while allowing optional integration of prior knowledge.

Despite these advances, CauFinder has practical limitations. As with other data‐driven causal inference approaches, unobserved variables may confound measured associations and influence inferred regulators. In practice, this risk is reduced when transcriptomic profiles provide sufficiently rich coverage of the molecular state (a causal information sufficiency assumption), as is often plausible for bulk RNA‐seq and single‐cell RNA‐seq. In addition, the network control process relies on prior interaction knowledge, which may be incomplete, context‐dependent, noisy, or partly correlational, and in CauFinder is used only to provide structural context for master‐regulator prioritization, whereas transition relevance is primarily determined by data‐derived causal weights. Accordingly, the network control results should be interpreted as prior‐guided regulator prioritization rather than a definitive reconstruction of the true control structure. Moreover, CauFinder nominates candidate causal regulators computationally, and definitive tests of necessity and sufficiency require experimental validation, for example using CRISPR‐based perturbations or Perturb‐seq. Although CRS showed prognostic value in TCGA lung cancer cohorts and predictive trends in in vitro TKI‐response settings, full clinical validation as a predictor of acquired EGFR‐TKI resistance will require uniformly TKI‐treated patient cohorts with annotated treatment exposure and outcome data.

In conclusion, CauFinder represents a practical framework for studying cell‐state or phenotype transitions and generating testable intervention hypotheses by integrating causal modelling and network control based on observed data. Future work may further expand its scope and robustness across broader biological settings.

## 4 Methods

### 4.1 Causal Disentangling Model

We consider a dataset $X={\left[x_{1},\text{…},x_{n}\right]}^{T}\in R^{n\times p}$, where each sample *x_i_* contains *p*features and was associated with an outcome *y_i_* representing a phenotype or state. The outcome *y_i_* can be binary, multi‐class, or continuous. This section describes the causal disentanglement module of CauFinder. Our goal was to identify transition‐relevant features by disentangling causal and spurious components underlying state or phenotype changes.

#### 4.1.1 Structural Causal Model

We analyze the mapping from input features *x*to the response variable *y*from a causal perspective. We first identify potential causal factors in the latent space and then map them back to causal features in the original space, which avoids directly modeling complex dependencies among input features. To this end, we construct a structural causal model (SCM) [76] in Figure 1B, where *x*  =  {*x^c^*,*x^s^*}denotes features in the original space and *z*  =  {*z^c^*,*z^s^*}denotes latent variables, with *y*representing the corresponding phenotype or state; (*x^c^*,*z^c^*) were causal components and (*x^s^*,*z^s^*) were spurious components. Under this hypothesis, both *z^c^* and *z^s^* were inferred from the observed data *x*. When estimating the causal effect of *z^c^* on *y*, a backdoor path $z^{c}\leftarrow x\to z^{s}\to y$ arises, where *x*acts as a common cause that induces a spurious association between *z^c^* and *y*via *z^s^*. To account for this confounding, we apply do‐calculus to identify the interventional effect *P*(*y*∣*do*(*z^c^*)). We then employ information flow $I(z^{c}\to y)$[77, 78] to quantify the causal influence of *z^c^* on model prediction *y*. This SCM was implemented using a dual autoencoder (Figure 1C).

#### 4.1.2 Dual Variational Autoencoder

Within the SCM framework, we implement the model using a dual variational autoencoder (DVAE) comprising a causal branch and a spurious branch. In the causal branch, input features *x* were gated by learnable weights *w*, yielding *x^w^* =  *x*⊙*w* (where ⊙ denotes element‐wise multiplication), which was encoded into the causal latent variable *z^c^* and reconstructed by a decoder. In parallel, the spurious branch applies complementary weights $\tilde{w}=1-w$ to obtain $x^{\tilde{w}}=x⊙\tilde{w}$, which was encoded into the spurious latent variable *z^s^* and reconstructed. Latent representations from both branches were jointly used to predict the sample state or phenotype *y*.

The DVAE was trained by minimizing the negative evidence lower bound (ELBO) loss,

(1) $$\;L_{\mathrm{ELBO}}=-E_{q\left(z|x\right)}\left(\log p(x|z\right)+\mathrm{KL}(q\left(z|x\right)||p\left(z\right)).\;$$

where *z* denotes the latent variable of the corresponding branch and KL(· ∥ ·) was the Kullback–Leibler divergence. For outcome prediction, we use a binary cross‐entropy (BCE) loss,

(2) $$\;L_{\mathrm{BCE}}=\frac{1}{n}\;\sum_{i=1}^{n}-\left(y_{i}\log{\hat{y}}_{i}+\left(1-y_{i}\right)\log\left(1-{\hat{y}}_{i}\right)\right),$$

where *y_i_* was the label of sample *i* and ${\hat{y}}_{i}$ was the predicted probability. When *y_i_* takes continuous values within [0,  1], it was treated as a soft label representing graded state assignment or transition propensity, and the same binary cross‐entropy formulation was used with soft targets. For multi‐class settings, BCE was replaced by categorical cross‐entropy.

#### 4.1.3 Causal Information Flow Measurement

Identifying potential causal factors in the latent space offers two advantages: (i) VAE latent variables were encouraged to be mutually independent, alleviating complex dependencies in the input space; and (ii) backdoor paths among latent variables were well‐defined, enabling principled control of confounding when measuring causal influence on prediction. We quantify the causal impact of *z^c^* on *y*using information flow $I(z^{c}\to y)$, which was the causal counterpart of mutual information MI(*z^c^*; *y*), and aim to maximize $I(z^{c}\to y)$ to isolate causal latent components.

Because *x* acts as a confounder between latent factors and the outcome, we compute the interventional distribution via the backdoor adjustment [14]:

(3) $$P\;\left(y|do\left(z^{c}\right)\right)=\sum_{x}P\left(y|z^{c},\;x\right)P\left(x\right)\;.$$

Intuitively, this formula estimates the causal effect of *z^c^* on *y* by considering different versions of *x* while keeping *z^c^* fixed. In causal theory, this approach, known as do‐calculus, effectively eliminates the influence of confounders by applying interventions to *z^c^*. The information flow was then defined by:

(4) $$\begin{array}{cc} & I\;\left(z^{c}\to y\right)\\ & =\int_{z^{c}}P\left(z^{c}\right)\sum_{y}P\left(y|do\left(z^{c}\right)\right)\log\frac{P\left(y|do\left(z^{c}\right)\right)}{\int_{z^{c}}P\left(z^{c}\right)P\left(y|do\left(z^{c}\right)\right)dz^{c}}dz^{c}\;. \end{array}$$

For a Detailed Derivation, See Note S8. We Maximize this Causal Information Flow by Minimizing

(5) $$\;L_{\mathrm{causal}}=-I\left(z^{c}\to y\right).$$

To ensure that *z^c^* captures the main predictive information about *y*, we introduce a fidelity loss that aligns predictions from *z^c^* with those from the full latent space:

(6) $$\;L_{\mathrm{fidelity}}=D_{KL}\;\left(p\left(y|z^{c}\right),\;p\left(y|z^{c},z^{s}\right)\right).$$

The validity of $I(z^{c}\to y)$ was established under the Causal Information Sufficiency Assumption (Note S9).

#### 4.1.4 Optimization Objectives and Training Strategy

Given the building modules described above, the learning of CauFinder can be formulated as minimizing the following overall loss L:

(7) $$L=\lambda_{1}\;L_{\mathrm{ELBO}}+\lambda_{2}L_{\mathrm{BCE}}+\lambda_{3}L_{\mathrm{causal}}+\lambda_{4}L_{\mathrm{fidelity}},$$

where $L_{\mathrm{ELBO}}$ was the negative evidence lower bound (ELBO) loss term that encourages faithful reconstruction while regularizing the approximate posterior toward the prior; $L_{\mathrm{BCE}}$ optimizes prediction of the state or phenotype *y*, $L_{\mathrm{causal}}$ aims to maximize the causal information flow from *z^c^* to *y*; $L_{\mathrm{fidelity}}$ ensures that *z^c^* captures the main information about *y*; λ_1_, λ_2_, λ_3_ and λ_4_ were designed to balance these loss terms. To stabilize optimization under multiple losses, we adopt a staged training strategy that emphasizes different loss components at different stages (Note S10); additional architecture details were provided in Note S11.

### 4.2 Quantifying Causal Contributions in the Original Space

After disentangling latent factors, we obtain an estimated transition‐relevant pathway $x\to z^{c}\to y$. To quantify feature‐level contributions in the original space, we compute SHapley Additive exPlanations (SHAP) scores for features *x_i_* based on the disentangled causal branch along this causal path, and define the causal weight *w_i_* as the normalized absolute SHAP value:

(8) $$\;w_{i}=\left|\mathrm{SHAP}\left(x_{i}\right)\right|/\sum_{j}\left|\mathrm{SHAP}\left(x_{j}\right)\right|\;.$$

Here, $w_{i}\in \left[0,1\right]$ reflects the relative causal importance of feature *x_i_* for the transition. Because SHAP primarily captures magnitude of contribution, we further compute gradients $\partial \hat{y}/\partial x_{i}$ to infer directionality, where a positive (negative) gradient indicates that increasing *x_i_* promotes (inhibits) the transition. We compute *w_i_* either globally across all samples or in a state‐specific manner (e.g., within state 0) to identify regulators relevant to a particular transition direction. Top‐ranked causal features were selected for downstream analysis and intervention.

### 4.3 Identification of Causal Master Regulators Based on Nonlinear Network Control

After estimating causal features and their weights, the downstream network control module prioritizes candidate master regulators that drive state or phenotype transitions by integrating causal weights with prior gene interaction networks. Specifically, we employ the Minimum Feedback Vertex Set (MFVS) method [16, 17] as a network‐informed prioritization step and integrate it with our causal weights.

#### 4.3.1 Prior Gene Interaction Network Construction

We construct an analysis‐specific prior gene interaction network to support downstream network control analysis and regulator prioritization. Following CEFCON, we adopt the NicheNet gene interaction network [79] as the prior, retaining intracellular signaling and gene regulatory interactions and excluding ligand–receptor edges to focus on within‐cell regulation. Undirected edges were treated as bidirectional. The original NicheNet network was defined using human gene symbols. For mouse data, we map genes using one‐to‐one orthologs from ENSEMBL and exclude ambiguous mappings, resulting in networks containing 25,332 genes and 5,290,993 edges for human, and 18,579 genes and 5,029,532 edges for mouse. To incorporate dataset‐specific interactions and improve network coverage, we further augment the prior network for each dataset by adding the top 1% of gene pairs identified via co‐expression analysis (Spearman correlation > 0.6). These data‐derived edges were used as supplementary structural information for downstream prioritization.

#### 4.3.2 Minimum Feedback Vertex Set Method for Identifying Master Regulators

We Adopt the Feedback Vertex Set (FVS) Framework [16, 17] for Nonlinear Network Control. Consider a Gene Regulatory Dynamical System

(9) $$\begin{array}{c} {\dot{s}}_{i}=F_{i}\;\left(s\right)=F_{i}\;\left(s_{i},\;s_{I_{i}}\right),\;i=1,\;2,\;\text{…},\;p,s.t.\;\frac{\partial F_{i}\left(s_{i},\;s_{I_{i}}\right)}{\partial s_{i}}<0. \end{array}$$

where *s_i_* denotes the expression level of gene *i* and *I_i_* denotes its set of regulatory predecessors. Following Zañudo et al. [80]., controlling all source nodes (in‐degree zero) together with a feedback node set that breaks all directed cycles was sufficient to steer the system to any of its attractors (cell states). Thus, we seek a minimal (or least‐cost) FVS while prioritizing nodes with higher causal weights *w_i_*. In this way, the weighted FVS framework combines structural information from the prior network with data‐derived causal weights.

We formulate this as a 0–1 integer linear program:

(10) $$\begin{array}{cc} & \min\sum_{i=1}^{p}u_{i}-\lambda u_{i}w_{i}s.t.\;r_{i}-r_{j}+pu_{i}\\ & \geq 1,\;\forall \;A_{ij}=1,r_{i}\in \left\{1,\;2,\;..,\;p\right\},\;\forall \;v_{i}\in V,\;u_{i}\in \left\{0,\;1\right\}, \end{array}$$

where *A* was the adjacency matrix of the directed network, *u_i_* indicates whether node *i* was selected into the FVS, and *r_i_* were auxiliary ordering variables. Because Equation (10) was NP‐hard, we apply standard graph reduction and solve the reduced ILP using the Gurobi optimizer. Candidate master regulators were not restricted to transcription factors and may include signaling or metabolic genes present in the prior network.

### 4.4 Counterfactual Generation for Causal state Transition

After identifying causal master regulators *x^c^* and the learned causal mapping *y*  = *f^c^* (*x^c^*), we generate counterfactual interventions to induce desired state transitions. Here, *f^c^* denotes the mapping from causal regulators *x^c^* to the outcome *y*via the causal latent variable *z^c^*. We formulate counterfactual generation as the following optimization problem:

(11) $$\arg\min_{{\tilde{x}}^{c}}\left\{{\left(f^{c}\left({\tilde{x}}^{c}\right)-\tilde{y}\right)}^{2}+\lambda d\left(x^{c},\;{\tilde{x}}^{c}\right)\right\},$$

where *x^c^* denotes the causal variables of a given sample, ${\tilde{x}}^{c}$ is the intervened (counterfactual) instance, $\tilde{y}\in \left\{0,1\right\}$ is the desired target state, and λ balances outcome matching and intervention cost. The distance function *d*(·, ·) penalizes deviations from the original instance, ensuring minimal perturbation. The objective yields counterfactual interventions that achieve the desired state transition with minimal changes to causal regulators.

#### 4.4.1 Path Controllability Score

To quantify the controllability of a sample during state transitions over a fixed number of iterations *T*, we define the path controllability score as:

(12) $$\mathrm{Path}\;\mathrm{Controllability}\;\mathrm{Score}\;=\frac{\mathrm{State}\;\mathrm{Change}}{\mathrm{Master}\;\mathrm{Regulators}\;\mathrm{Change}}\;.$$

Here, the state change is defined as the difference between the final state at iteration *T* and the initial state, while the master regulators change denotes the cumulative change in master regulators over *T* iterations. A higher score indicates that a sample achieves a larger state transition with smaller perturbations to master regulators, corresponding to higher controllability.

### 4.5 Datasets and Data Preprocessing

Bulk LUAS and Watermelon‐PC9 scRNA‐seq datasets were obtained from published studies [61, 64, 68]. Key preprocessing steps (quality control thresholds, highly variable gene selection, and cell grouping) followed the original protocols and were summarized in Note S12.

### 4.6 Cell Culture

PC9 human lung adenocarcinoma cells were obtained from Shanghai Zhongqiao Xinzou Biotechnology Co., Ltd. (Shanghai, China), authenticated by STR profiling, and confirmed to be mycoplasma‐free. Cells were maintained in Dulbecco's Modified Eagle Medium (DMEM) supplemented with 10% fetal bovine serum (FBS) and 1% penicillin/streptomycin at 37°C in a humidified incubator containing 5% CO_2_.

### 4.7 SiRNA Transfection

PC9 cells were seeded in 6‐well plates and transfected with negative control siRNA or *DAAM1*‐targeting siRNAs using the indicated siRNA transfection reagent according to the manufacturer's instructions. Two independent *DAAM1*‐targeting siRNAs synthesized by Sangon Biotech were used. The siRNA sequences were as follows: siDAAM1‐1 sense, 5′‐GCGAAAUGAUAGCAACUUUdTdT‐3′, and antisense, 5′‐AAAGUUGCUAUCAUUUCGC dTdT‐3′; siDAAM1‐2 sense, 5′‐ACUACUAGAUAGAAUUAUAdTdT‐3′, and antisense, 5′‐UAUAAUUCUAUCUAGUAGUdTdT‐3′. After transfection, cells from the same experimental batch were used for Western blot‐based knockdown validation and CCK‐8 basal viability and osimertinib dose‐response assays.

### 4.8 Western Blotting

For *DAAM1* knockdown validation, transfected PC9 cells were harvested and lysed for total protein extraction. Equal amounts of protein were separated by SDS‐PAGE and transferred onto membranes. Membranes were blocked and incubated overnight at 4°C with a rabbit polyclonal anti‐DAAM1 antibody (Proteintech, Cat. No. 14876‐1‐AP; 1:2000). The membranes were then incubated with an HRP‐conjugated goat anti‐rabbit IgG H&L secondary antibody (Abcam, Cat. No. ab6721; 1:5000), and DAAM1 protein bands were detected using chemiluminescence.

After DAAM1 detection, membranes were stripped to remove bound antibodies, washed extensively, and reprobed with a rabbit polyclonal anti‐GAPDH antibody (ZENBIO, Cat. No. 380626; 1:5000), followed by the same HRP‐conjugated goat anti‐rabbit IgG H&L secondary antibody (Abcam, Cat. No. ab6721; 1:5000). GAPDH was used as the loading control. *DAAM1* knockdown was assessed by comparing DAAM1 protein levels in *DAAM1* siRNA‐transfected cells with those in negative control siRNA‐transfected cells.

### 4.9 CCK‐8 Basal Viability and Osimertinib Dose‐Response Assays

Following siRNA transfection, PC9 cells were reseeded into 96‐well plates. For basal viability analysis, siRNA‐transfected cells were cultured with vehicle control (DMSO), and viability was measured at Day 0, Day 2, Day 4, and Day 6. Blank‐subtracted values were normalized to Day 0 to generate relative viability curves.

For osimertinib dose‐response assays, siRNA‐transfected cells were treated with osimertinib (MCE, HY‐15772) at 0, 1, 2, 3, 5, 10, 50, and 100 nM for 72 h. Blank‐subtracted values were normalized to the vehicle‐treated control for dose‐response analysis.

For both assays, cell viability was measured using the CCK‐8 Cell Counting Kit (Vazyme, Cat. No. A311‐01), and absorbance at 450 nm was recorded with a SpectraMax iD5 microplate reader (Molecular Devices, San Jose, CA, USA).

### 4.10 IC_50_ Fitting and Statistical Analysis

Dose‐response curves were fitted in GraphPad Prism version 11.0.0 (84) using the built‐in nonlinear regression model, log(inhibitor) versus normalized response with variable slope. Cell viability values were normalized to the corresponding vehicle‐treated condition. For log‐scale visualization, the 0 nM vehicle condition was plotted at a nominal x‐axis position of 0.1 nM, while its measured response was retained as the vehicle baseline. Fitted IC_50_ values were calculated from each independent biological repeat.

Four independent biological repeats were performed for each *DAAM1* siRNA condition. Fitted IC_50_ values from independent biological repeats were compared between negative control and *DAAM1* knockdown groups using a two‐tailed unpaired t‐test with Welch's correction in GraphPad Prism. Data were presented as mean ± SD unless otherwise indicated, and *p* < 0.05 was considered statistically significant.

### 4.11 AI Statement

Artificial intelligence tools (ChatGPT, OpenAI) were used solely for language editing. All scientific content was produced and validated by the authors.

## Author Contributions

Conceptualization, **L.C**., **K.A**., and **J.L**.; Methodology, **C.Z**., **Z.C**., **Y.M**., and **W.G**.; Software, **C.Z**., **Z.C**., and **Y.M**.; Formal analysis, **Y.M**., **Z.C**., and **C.Z**.; Investigation, **C.Z**., **Z.C**., **Y.M**., **Z.S**., **D.C**., **S.T**., and **Y.X**.; Validation, **Z.S**.; Visualization, **Y.M**., **Z.C**., and **C.Z**.; Supervision, **L.C**., **K.A**., and **J.L**.; Writing – original draft, **C.Z**., **Z.C**., **Y.M**., and **Z.S**.; Writing – review & editing, **C.Z**., **Z.C**., **Y.M**., **Z.S**., **H.J**., **J.L**., **K.A**., and **L.C**.

## Conflicts of Interest

The authors declare no conflicts of interest.

## Supporting information




**Supporting File**: advs76177‐sup‐0001‐SuppMat.pdf.

## Data Availability

All the datasets analyzed in this study are publicly available. The prior gene interaction network was derived from NicheNet [79], which can be downloaded from https://github.com/saeyslab/nichenetr. Human and mouse embryonic stem cell single‐cell RNA‐seq datasets are available in the Gene Expression Omnibus (GEO) under accession codes GSE75748 (https://www.ncbi.nlm.nih.gov/geo/query/acc.cgi?acc = GSE75748) and GSE81682 (https://www.ncbi.nlm.nih.gov/geo/query/acc.cgi?acc = GSE81682), respectively. The cell conversion datasets were obtained from the CellNet [81] data resource and are available at Zenodo (https://doi.org/10.5281/zenodo.18857326). TCGA datasets used in this study can be downloaded from the UCSC Xena browser at https://xenabrowser.net/datapages/. Human Astrocytoma datasets are available in the NODE database under project accession number OEP001032 (https://www.biosino.org/node/sample/detail/OES032241). Mouse Astrocytoma datasets can be found in the NODE database under project accession number OEP002019 (https://www.biosino.org/node/project/detail/OEP002019). Single cell RNA‐seq data for Watermelon system cells used in both cycling persisters and drug resistance studies, are available under GEO accession code GSE150949 (https://www.ncbi.nlm.nih.gov/geo/query/acc.cgi?acc = GSE150949). The data on drug responses to EGFR signaling pathway targeted therapies in individual patients and bulk RNA sequencing are derived from two databases: Genomics of Drug Sensitivity in Cancer [70] (GDSC) and Cancer Cell Line Encyclopedia [69] (CCLE). The GDSC data can be accessed and downloaded from (https://www.cancerrxgene.org/downloads/drug_data), while the CCLE data are available from (https://sites.broadinstitute.org/ccle/datasets). The Colorectal Cancer Liver dataset is sourced from website [73] (http://www.cancerdiversity.asia/scCRLM).
