# Supplementary material for: CauFinder: Steering Cell‐State and Phenotype Transitions by Causal Disentanglement Learning
Source: Adv Sci (Weinh). 2026 Jun 16:e76177. Online ahead of print. doi: 10.1002/advs.76177 (PMC13336959; doi:10.1002/advs.76177)
Supplement: Supplementary file 1 — Supporting File: advs76177‐sup‐0001‐SuppMat.pdf. [file ADVS-9999-e76177-s001.pdf]

Supplementary Information for

CauFinder: Steering cell-state and phenotype transitions by causal disentanglement learning

Chengming Zhang, Zexi Chen, Yuanxiang Miao, Zuolin Shen, Deyu Cai, Shijie Tang, Yun Xue, Weifeng Guo, Hongbin Ji, Jian Liu, Kazuyuki Aihara, and Luonan Chen

Correspondence: Luonan Chen, Kazuyuki Aihara, Jian Liu
Email: lnchen@sibcb.ac.cn (L.C.), kaihara@g.ecc.u-tokyo.ac.jp (K.A.), JianL@intl.zju.edu.cn (J.L.)

This PDF file includes:

Notes S1 to S12
Figures S1 to S21
Tables S1 to S2
SI References

Supplementary notes

Note S1. Simulation dataset construction

To systematically evaluate CauFinder in different causal inference scenarios, we constructed two types of simulated datasets:(1) Synthetic Simulation Data, designed to explore the effects of causal and spurious relationships in an idealized setting, and (2) Perturbation-based Simulation Data, generated using CausalRegNet[1], a model designed to simulate gene perturbation experiments and better reflect real-world gene expression variability. We describe each dataset below:

Synthetic simulation data. The simulated dataset was constructed to explore the effects of both causal and spurious relationships among observed variables. In this simulation, we focus on three types of observed variables: x^c, x^s, and y. The variables x^c have a direct causal effect on the outcome y, while the spurious variables x^s are correlated with$\mathrm{y}$ without having a direct causal effect. Additionally, the outcome variable y is directly observed. The unobserved variable $u \sim N(\mu^u, \sigma^u)$ acts as a confounder, creating dependencies among the observed variables. The primitive variable $c \sim N(\mu^c, \sigma^c)$ directly influences the observed variables with a causal effect.

The spurious variables x^s are generated by the function $f_1(u)$. Although x^s has no direct causal effect on the outcome variable y, it is correlated with y through the shared dependence on u. The observed variables with a causal effect on y, denoted as x^c, are generated by combining the effects of $f_2(x^s)$ and $g_1(c)$. This means that some x^c can be influenced by both partial spurious variables and the primitive causal variable, reflecting the complexity often observed in real-world data. The outcome variable y is modeled as a composite of multiple effects. Specifically, y is expressed as $\lambda g_2(x^c) + (1-\lambda) f_3(u) + \gamma\epsilon$, where λ controls the strength of the causal effect from x^c, γ controls the noise scale, and ϵ is a non-Gaussian noise to ensure the directed causal relation[2] from x to y. In this construction, λ plays a crucial role in balancing the contributions of x^c and u to y. When λ is close to 1, the causal effect of x^c dominates, whereas when λ is close to 0, the effect of the unobserved variable u is more prominent. The noise term $\gamma\epsilon$ adds randomness to y, simulating the inherent variability observed in real-world data.

In the linear scenario, f_1, f_2, f_3, g_1, g_2 are all functions where a random weight is multiplied by the input. In the nonlinear scenario, the input is further transformed using the hyperbolic tangent function. The sample size is set to 200, with 90 confounding features (x^s) and 10 causal features (x^c).

Perturbation-based simulation data. To better reflect real-world gene expression variability, we generated perturbation-based simulated single-cell data using a two-step process based on the CausalRegNet framework. CausalRegNet, a multiplicative effect structural causal model, was first used to simulate gene perturbation experiments. Briefly, CausalRegNet generates both observational (unperturbed) and interventional (perturbed) gene expression profiles.

In our simulations, we generated data for 100 genes, with 10 genes randomly selected as targets for perturbation. The phenotypic outcome y, was modeled as a function of these genes, specifically $y = f(x)$ where x represents the expression levels of these genes, and $f(.)$ is a function that multiplies the input by a random weight. This simulates a scenario where the phenotype is directly influenced by the perturbed genes.

Note S2. Benchmark methods and software programs

In our benchmark experiments, we employed several methods to evaluate the extraction of feature subsets with causal effects on the state variable y from all observed variables. These methods can be categorized into three groups: causal inference methods, deep learning models, and machine learning models.

Causal model: we used the PC algorithm[3], which constructs a directed causal network based on the input variables, focusing only on those that have a causal effect on y to ensure a relevant comparison. In addition, we included several modern causal discovery methods, including NOTEARS[4], FCI[5], and LINGAM[6]. These methods infer causal graph structures under different assumptions. We also evaluated GES[7] as a score-based causal discovery method, but it was not included in the final benchmark because it was not computationally tractable under our high-dimensional transcriptomic setting. To adapt causal

graph-learning methods to our task, we treated y as a target node and ranked features based on their inferred relationships with y.

Deep learning models: We used the variational autoencoder (VAE), implemented in PyTorch (v2.1.2)[8]. For the VAE model, we set the number of hidden neurons to 64 and the latent space variables to 10. Similar to CauFinder, we used the latent space to predict y during training. We assigned weights to each feature using SHAP values (VAE_SHAP) or by calculating the gradient of each feature (VAE_Grad). This approach can be treated as an ablation study of CauFinder since it lacks the causal component.

Machine learning models: Implemented using Python's statistic machine learning libraries such as scikit-learn (v1.13)[9], we used T-test, random forest (RF), mutual information (MI), Pearson correlation coefficients (PCC), and Spearman correlation coefficients (SCC). For the Random Forest model, we set the number of trees to 50 and the maximum depth to 2. Causal features were selected based on computed feature importance.

Additionally, we used the following driver identification and GRN inference methods for benchmarking on real datasets. These GRN inference methods were originally developed to infer gene–gene regulatory relationships or dynamic regulatory networks, rather than to directly prioritize regulators for state-transition classification. Therefore, we adapted their outputs to a regulator-ranking framework by incorporating the state variable y when applicable and ranking genes based on their inferred relationships or edge weights with respect to y. These methods were interpreted as cross-paradigm references.

CellOracle: CellOracle[10] utilizes single-cell RNA sequencing (scRNA-seq) data and a prior base gene regulatory network (GRN), or a combination of scRNA-seq and single-cell ATAC sequencing (scATAC-seq) data to compute the base GRN. Based on this base GRN, it constructs cell type-specific GRNs and employs various strategies to identify key transcription factors (TFs). These key TFs are then subjected to simulated perturbations to predict changes in cell states. In our comparisons, we followed the strategy outlined in CEFCON, using the default degree centrality of TFs in the GRN to measure their importance.

CEFCON: CEFCON[11] is a network framework for inferring gene regulatory relationships and characterizing their dynamic properties from the perspective of network control theory, aimed at identifying driver regulatory factors in cell fate decisions. CEFCON takes prior gene interaction networks and scRNA-seq data as inputs and combines network attention coefficients, minimum dominating set (MDS), and minimum feedback vertex set (MFVS) methods to obtain a final influence score for each gene. This score serves as the criterion for determining whether a gene is a driver of state transitions.

WMDS.net: WMDS.net[12] is a weighted minimum dominating set network model based on structural controllability theory. It identifies the minimum dominating set of driver nodes in a transcriptional co-expression network as critical drivers for network state transitions. WMDS.net combines the node degree and the significance of differential co-expression of genes between two states to measure node controllability within the transcriptional network. Notably, CEFCON can only be applied to datasets with actual gene names, as it relies on known gene interaction networks for regulatory inference.

GENIE3: GENIE3[13] is a tree-based approach that employs Random Forest or Extra-Trees to infer gene regulatory interactions. Each gene's regulatory influence is ranked based on predictive importance. To integrate GRN inference methods with our aim, we incorporated the sample state y as an additional feature into the expression matrix during implementation. The driver genes were selected based on those connected to y in the inferred GRN network, with edge weights serving as gene importance metrics.

Velorama: Velorama[14] uses RNA velocity and pseudotime to infer causal gene regulatory interactions via Granger causal inference, distinguishing between fast and slow regulators. For simulated data, we normalized the cellular state y as pseudotime input. In real datasets, we utilized scVelo[15] computed pseudotime values with expression matrices as method inputs. The mean weights of edges associated with each gene in the inferred GRN were subsequently calculated as gene importance scores.

SCODE: SCODE[16] is a matrix factorization-based approach using ordinary differential equations (ODEs) to infer time-dependent gene regulatory networks from single-cell differentiation data. In our implementation, all genes were predefined as transcription factors, with scVelo caculated pseudotime values as inputs. Subsequent gene importance calculations followed the same protocol as employed in Velorama.

Mogrify: Mogrify[17] is a computational framework for predicting transcription factors required for direct cell conversion between defined source and target cell types. It integrates differential expression with regulatory network information to estimate transcription factor regulatory influence and prioritize candidate conversion factors. Mogrify is designed for transcription factor–based cell reprogramming rather than general causal feature discovery. We therefore included it as a complementary reference method and performed result-level comparisons using the published human cell conversion cases and reported regulator sets, as no publicly available reproducible implementation was identified.

Note S3. Evaluation criteria on benchmark datasets

For the evaluation of our models on benchmark datasets, we employed a variety of metrics to ensure comprehensive performance assessment. These metrics include:

Accuracy (ACC): The proportion of true results (both true positives and true negatives) among the total number of cases examined. It is calculated as $\frac{TP+TN}{TP+TN+FP+FN}$, where TP is true positives, TN is true negatives, FP is false positives, and FN is false negatives.

Area Under the Receiver Operating Characteristic Curve (AUC): Measures the ability of the model to distinguish between classes. A higher AUC value indicates better model performance. The AUC ranges from 0 to 1, where 1 represents a perfect model and 0.5 represents a model with no discriminative ability.

Recall (Sensitivity or True Positive Rate): The proportion of actual positives correctly identified by the model. It is calculated as $\frac{TP}{TP+FN}$. High recall indicates that the model is able to identify most of the positive cases (causal features).

Specificity (True Negative Rate): The proportion of actual negatives correctly identified by the model. It is calculated as $\frac{TN}{TN+FP}$. High specificity indicates that the model is able to identify most of the negative cases (spurious features).

Precision (Positive Predictive Value): The proportion of positive identifications that are actually correct. It is calculated as $\frac{TP}{TP+FP}$. High precision indicates that the model has a low false positive rate.

F1 Score: The harmonic mean of precision and recall, providing a balance between the two. It is calculated as $F1 = 2\frac{\text{Precision.Recall}}{\text{Precision+Recall}}$. This metric is particularly useful when the class distribution is imbalanced.

Matthews Correlation Coefficient (MCC): A correlation coefficient that takes into account true and false positives and negatives, providing a balanced measure even if the classes are of very different sizes. It is calculated as $MCC = \frac{TP.TN-FP.FN}{\sqrt{(TP+FP)(TP+FN)(TN+FP)(TN+FN)}}$. The MCC value ranges from -1 to +1, where +1 indicates a perfect prediction, 0 indicates no better than random prediction, and -1 indicates total disagreement between prediction and observation.

These metrics provide a comprehensive understanding of the model's performance, highlighting strengths and potential areas for improvement.

Note S4. Choosing the number of causal drivers

Determining the appropriate threshold to identify the number of causal drivers is challenging. Typically, CauFinder assigns a causal weight to each feature, with higher values indicating a greater likelihood of being a causal driver.

For evaluating performance on simulated data, we explored two methods. The first method involves selecting the top 10 features as causal drivers. The second method involves ranking the features by their causal weights in descending order and then summing these weights cumulatively, selecting the top 30% of features based on this cumulative proportion. For clarity and alignment with the simulated data's construction, which includes 10 causal features, we opted to use the first method, selecting the top 10

features as causal drivers for all benchmark methods. In real-world scenarios, we combine prior knowledge and control theory to assist in this selection.

Note S5. Ablation study on model components

To evaluate the contribution of individual components within CauFinder, we conducted an ablation study, systematically removing key elements and assessing the impact on performance. Specifically, we focused on the causal disentanglement losses, $\mathcal{L}_{\text{causal}}$ and $\mathcal{L}_{\text{fidelity}}$, which are designed to ensure that the learned latent representations effectively separate causal and non-causal factors and encourage that these representations reflect the true data distribution.

We compared the following four variants of CauFinder: (1) CauFinder (Full Model): The complete model with all loss components and staged training. (2) CauFinder w/o Causal (Removing $\mathcal{L}_{\text{causal}}$ only): This variant excluded the causal loss term ($\mathcal{L}_{\text{causal}}$), while retaining the fidelity loss ($\mathcal{L}_{\text{fidelity}}$) and staged training. (3) CauFinder w/o Fidelity (Removing $\mathcal{L}_{\text{fidelity}}$ only): This variant excluded the fidelity loss term ($\mathcal{L}_{\text{fidelity}}$), while retaining the causal loss ($\mathcal{L}_{\text{causal}}$) and staged training. (4) CauFinder w/o Staged Training: This variant utilized both loss terms ($\mathcal{L}_{\text{causal}}$ and $\mathcal{L}_{\text{fidelity}}$) but removed the staged training procedure.

All model variants were trained and evaluated on the same simulated datasets and using the same evaluation metrics (AUC) as described in the main experiments. This allowed for a direct comparison of the performance impact of each ablated component. We hypothesized that removing any of these components would lead to deterioration in performance, indicating their importance for achieving accurate causal feature identification.

Note S6. Extended analysis of state transitions in lung cancer (PC9) and colorectal cancer

To assess CauFinder's robustness, we constructed four input settings: (1) day14 cycling persisters paired with day0 lineage ancestors; (2) day14 cycling persisters paired with day7 lineage ancestors; (3) day0 cycling vs. non-cycling persisters; and (4) day14 cycling vs. non-cycling persisters. Despite different inputs and random seeds, CauFinder consistently identified high-weighted drivers related to ROS and fatty acid metabolism (FAM). Gene Set Enrichment Analysis (GSEA) using KEGG pathways confirmed that ROS- and FAM-related genes exhibited high weights, with enrichment in associated carbon metabolism pathways (Fig. S15B-F).

We explored the correlation between the expression of candidate causal drivers and the IC_{50} of four EGFR-targeting drugs in the CCLE and GDSC datasets. Beyond the top-ranked drivers (*CD9*, *BARD1*, *SLC1A5*), significant correlations ($P < 0.05$, Pearson correlation) were found for genes including *TFDP1*, *RRBP1*, *ANXA2*, and *DAAM1* with the sensitivity of one or more drugs. The Causal Drug Response Score (CRS) provided a more robust metric than single genes. In the GDSC dataset, CRS correlated with IC_{50} ($P = 0.006$); in the CCLE dataset, sensitive cell lines ($IC_{50} \leq 8$) showed significantly higher CRS than resistant ones ($IC_{50} > 8$), supporting the score's discriminative power in cell-line drug-response settings.

We utilized the path controllability score to evaluate forward (day0/7 to day14) and reverse transitions. Models initiating from day7 showed significantly higher path controllability scores than those from day0 ($P = 0.0407$ for increasing, $P = 0.0439$ for decreasing, Kolmogorov-Smirnov test). Specific analysis of pseudo-cell 4 in the day0 group revealed a higher controllability score (0.044794) compared to its peers, with differential expression analysis identifying genes such as *POLR3A* and *COX19* as potential regulators. In simulations of drug sensitivity, while forward transitions from sensitive to resistant states were efficiently modeled using drivers like *DAAM1* and *SLC1A5*, the simulated reverse shift toward sensitivity showed more complex transition paths, suggesting higher barriers to reversing established drug resistance.

To train the CauFinder model on the spatial datasets, we constructed specific paired-state inputs representing the transition between normal and malignant states. For the primary colorectal cancer (P1) sample, we explicitly selected the annotated tumor tissue clusters and normal mucosa tissue clusters as the paired input. For the liver metastasis (LM1) sample, to capture the interaction at the invasive front, we selected two specific cell clusters located at the tumor-liver interface: Cluster 1 (representing the tumor side

of the interface) and Cluster 6 (representing the liver tissue side). These pairs were used to infer causal drivers and simulate state transitions.

Note S7. Pseudo-cell construction

To address the sparsity and high dimensionality of single-cell and spatial transcriptomics data, pseudo-cells were generated to retain dominant state-level transcriptional signals while reducing computational burden, following a standardized workflow: within-state reclustering was performed for each predefined biological state (e.g., day0 initial state, drug-sensitive state) using the same algorithm as the primary dataset analysis (MCGAE algorithm for spatial transcriptomics data, standard Louvain clustering for single-cell RNA-seq data); mean gene expression values were calculated for each sub-cluster derived from reclustering; each sub-cluster, represented by its mean expression profile, was designated as a single pseudo-cell.

For dataset-specific applications: The lung cancer cycling persister cell dataset's three-state time-series data (day0, day7, day14) was converted into 13 pseudo-cells, including 5 representing initial states (pooled day0 and day7 lineages) and 8 representing the day14 target state; drug-sensitive and resistant states were converted into 4 and 6 pseudo-cells, respectively.

In the primary colorectal cancer (P1) spatial dataset, pseudo-cells were generated using the identical workflow as the lung cancer dataset. Key characteristics included: connective tissue-derived pseudo-cells showing strong potential for transitioning to a cancerous state; a pseudo-cell representing a mixed population of connective tissue and tumor cells (excluded from model training) that was predicted to shift toward a normal state but not a tumor state; boundary-region pseudo-cells showing predicted state shifts upon modulation of high-SHAP-value causal drivers (*IGKC* and *NFKBIA*); and tumor-derived pseudo-cells predicted to shift toward a normal state via two previously validated anti-tumor gene combinations.

For the liver metastasis (LM1) spatial dataset, interface cells (tumor-liver boundary) were first assigned to 4 clusters via MCGAE clustering, then converted into pseudo-cells following the standard workflow. Notable features included: pseudo-cells showing tissue specificity (proximal to tumor or liver tissue) consistent with manual spatial annotation; intermediate pseudo-cells (shared between tumor and liver-adjacent clusters) exhibiting strong tumor transition potential but no predicted transition to liver-like transcriptional states (reflecting the metastatic origin of LM1, not primary liver cancer); and interface pseudo-cells predicted to shift toward a normal state via modulation of candidate drivers (e.g., *ITGA6* and *NOTCH1*), with specific driver combinations producing varying degrees of predicted state shifts reflecting the balance between pro- and anti-invasive signals.

Note S8. Derivation of the estimator of information flow

This section provides the detailed derivation of the estimator for the causal information flow $I(z^c \to y)$.

The causal information flow between the causal factors z^c and the prediction y can be calculated as:

$$I(z^c \to y) = \int_{z^c} P(z^c) \sum_y P\big(y|do(z^c)\big) \log \frac{P\big(y|do(z^c)\big)}{\int_{z^c} P(z^c) P\big(y|do(z^c)\big) dz^c} dz^c. \tag{S1}$$

Expanding this, we have:

$$\begin{aligned} I(z^c \to y) = &\int_{z^c} P(z^c) \left(\sum_y P\big(y|do(z^c)\big) \log P\big(y|do(z^c)\big) \right) dz^c \\ &- \sum_y \int_{z^c} P(z^c) P\big(y|do(z^c)\big) dz^c . \log \int_{z^c} P(z^c) P\big(y|do(z^c)\big) dz^c. \end{aligned} \tag{S2}$$

Next, we compute $P\big(y|do(z^c)\big)$, which can be efficiently estimated using Monte Carlo sampling. Specifically, we have:

$$P(y|do(z^c)) = \sum_x P(y|z^c, x)P(x) = \sum_x \int_{z^s} P(z^s|z^c, x)P(y|z^c, z^s)P(x)dz^s$$
$$\approx \frac{1}{N^x N^s}\sum_{k=1}^{N^x}\sum_{j=1}^{N^s} P(y|z^c, z^s_{kj}). \tag{S3}$$

where k indexes the N^x samples x_k drawn from the dataset, and j indexes the N^s samples for each x_k, i.e., $z^s_{kj} \sim P(z^s|z^c, x_k)$. Here, we approximate the true posterior distribution $P(z^s|z^c, x_k)$ using the variational distribution $q(z^s|z^c, x_k)$. Note that in Equation (S3), x, z^c, and z^s do not necessarily belong to the same sample from the original dataset. Then, we have

$$\int_{z^c} P(z^c)P(y|do(z^c))dz^c$$
$$= \int_{z^c}\sum_x \int_{z^s} P(x)P(z^c)P(z^s|z^c, x)P(y|z^c, z^s)dz^s\, dz^c$$
$$\approx \frac{1}{N^c N^x N^s}\sum_{i=1}^{N^c}\sum_{k=1}^{N^x}\sum_{j=1}^{N^s} P(y|z^c_i, z^s_{kj}). \tag{S4}$$

Similarly, i indexes the N^c samples from z^c's marginal distribution, i.e., $z^c_i \sim P(z^c)$, k indexes the N^x samples from x's marginal distribution $P(x)$, and j indexes the N^s samples of z^s for each pair (z^c_i, x_k), i.e., $z^s_{ikj} \sim P(z^s|z^c_i, x_k)$. In practice, we approximate the true posterior distribution $P(z^s|z^c_i, x_k)$ using the variational distribution $q(z^s|z^c_i, x_k)$. Combining these, we get:

$$I(z^c \to y) = \frac{1}{N^c}\sum_{i=1}^{N^c}\sum_y \left(\frac{1}{N^x N^s}\sum_{k=1}^{N^x}\sum_{j=1}^{N^s} P(y|z^c_i, z^s_{kj})\right)\log\left(\frac{1}{N^x N^s}\sum_{k=1}^{N^x}\sum_{j=1}^{N^s} P(y|z^c_i, z^s_{kj})\right)$$
$$-\sum_y \left(\frac{1}{N^c N^x N^s}\sum_{i=1}^{N^c}\sum_{k=1}^{N^x}\sum_{j=1}^{N^s} P(y|z^c_i, z^s_{kj})\right).\log\left(\frac{1}{N^c N^x N^s}\sum_{i=1}^{N^c}\sum_{k=1}^{N^x}\sum_{j=1}^{N^s} P(y|z^c_i, z^s_{kj})\right)$$
$$= \frac{1}{N^c N^x N^s}\left[\sum_{i=1}^{N^c}\sum_y \left(\sum_{k=1}^{N^x}\sum_{j=1}^{N^s} P(y|z^c_i, z^s_{kj})\right).\log\left(\frac{1}{N^x N^s}\sum_{k=1}^{N^x}\sum_{j=1}^{N^s} P(y|z^c_i, z^s_{kj})\right)\right. \tag{S5}$$
$$\left.-\sum_y \left(\sum_{i=1}^{N^c}\sum_{k=1}^{N^x}\sum_{j=1}^{N^s} P(y|z^c_i, z^s_{kj})\right).\log\left(\frac{1}{N^c N^x N^s}\sum_{i=1}^{N^c}\sum_{k=1}^{N^x}\sum_{j=1}^{N^s} P(y|z^c_i, z^s_{kj})\right)\right].$$

Note S9. Impact of unobserved variables on information flow

In our framework, we initially assume that all variables x are observed. However, a natural question arises: what happens if some variables are unobserved? This situation is common in real-world data, as it is often impossible to observe every variable. To address this, we refined our division of features in both the latent and original spaces, by introducing u to represent unobserved variables (Figure S21). We further split the original z^c into z^c_{xu}, representing the causal latent space shared by x and u, and z^c_x, specific to x. Additionally, z^c_u denotes the causal latent space unique to u. Consistent with our previous notation, $z^c = \{z^c_{xu}, z^u_x\}$. In this context, the causal latent space comprises both z^c and z^c_u. A similar approach is applied to defining spurious latent spaces. Under this setting, we formalize the Causal Information Sufficiency Assumption, which states that if x contains all essential causal information about y, the influence of unobserved variables u on the causal information flow $I(z^c \to y)$ is minimized.

The core of our exploration focuses on the impact of unobserved variables u on the causal information flow $I(z^c \to y)$. This inquiry centers on u's effect on $P(y|do(z^c))$, the conditional distribution given the

intervention on z^c using do-calculus. From a probabilistic graphical perspective, compared to the case where uu is absent, four additional paths are introduced: $u \to z_{xu}^c; u \to z_u^c;\ u \to z_{xu}^s; u \to z_u^s$. Although there are $2^4 - 1$possible combinations of the presence and absence of these latent variables, the primary distinction lies in whether z_u^c is present or absent.

Therefore, we focus our discussion on these two scenarios: when z_u^c is present and when z_u^c is absent. We consider two scenarios: when z_u^c is present and when z_u^c is absent.

(1) When z_u^c is present, the causal structure model can be represented as: $x, u \to z_{xu}^c; x, u \to z_{xu}^s; x \to z_x^c; x \to z_x^s; u \to z_u^c;\ u \to z_u^s; z_u^c, z^c, z^s \to y$. In this case, the conditional distribution $P(y|do(z^c))$ is derived as follows:

$$
\begin{aligned}
P(y|do(z^c)) &= \sum_{x,u} P(y|z^c, x, u)P(x, u) \\
&= \sum_{x,u} \int_{z^s} \int_{z_u^s} \int_{z_u^c} P(z^s|x, u)P(z_u^c|u)P(z_u^s|u)P(y|z^c, z^s, z_u^c)P(x, u)\, dz^s dz_u^s dz_u^c \\
&= \sum_{x,u} \int_{z^s} \int_{z_u^c} P(z^s|x)P(z_u^c|u)P(y|z^c, z^s, z_u^c)P(u|x)P(x)\, dz^s dz_u^c \\
&= \sum_{x} \int_{z^s} P(z^s|x)P(x) \left(\sum_{u} \int_{z_u^c} P(y|z^c, z^s, z_u^c)\, P(u|x) dz_u^c \right) dz^s \\
&= \sum_{x} \int_{z^s} P(z^s|z^c, x)\tilde{P}(y|z^c, z^s)P(x) dz^s \\
&\approx \frac{1}{N^x N^s} \sum_{k=1}^{N^x} \sum_{j=1}^{N^s} \tilde{P}(y|z^c, z_{kj}^s).
\end{aligned}
\tag{S6}
$$

Here, $\tilde{P}(y|z^c, z^s) = \sum_u \int_{z_u^c} P(y|z^c, z^s, z_u^c)\, P(u|x) dz_u^c$represents the average effect of z_u^c on y. We assume that $P(z^s|x, u) = P(z^s|x)$, leveraging the conditional independence assumption. This means that x contains all the necessary information about z^s, and thus the influence of u on z^s can be ignored. Since u is unobserved, this probability is not directly computable, making the calculation of $I(z^c \to y)$ potentially inaccurate in this scenario.

(2) When z_u^c is absent, the causal structure model can be represented as: $x, u \to z_{xu}^c; x, u \to z_{xu}^s; x \to z_x^c; x \to z_x^s; u \to z_u^c;\ u \to z_u^s; z_u^c, z^c, z^s \to y$. In this case, the conditional distribution $P(y|do(z^c))$ is derived as follows:

$$
\begin{aligned}
P(y|do(z^c)) &= \sum_{x,u} P(y|z^c, x, u)P(x, u) = \sum_{x,u} \int_{z^s} \int_{z_u^s} P(z^s|x, u)P(z_u^s|u)P(y|z^c, z^s)P(x, u)\, dz^s dz_u^s \\
&= \sum_{x,u} \int_{z^s} P(z^s|x, u)P(y|z^c, z^s)P(x, u) dz^s \\
&= \sum_{x} \int_{z^s} P(z^s|z^c, x)P(y|z^c, z^s)P(x) \left(\sum_{u} P(u|x) \right) dz^s \\
&= \sum_{x} \int_{z^s} P(z^s|z^c, x)P(y|z^c, z^s)P(x) dz^s \\
&\approx \frac{1}{N^x N^s} \sum_{k=1}^{N^x} \sum_{j=1}^{N^s} P(y|z^c, z_{kj}^s).
\end{aligned}
\tag{S7}
$$

This demonstrates that, in this scenario, the presence of unobserved variables u does not affect $P(y|do(z^c))$, and therefore, does not impact the defined causal information flow $I(z^c \to y)$.

In conclusion, our analysis confirms that the definition of $I(z^c \to y)$ holds accurate when x captures all essential causal information concerning y. This condition is typically met in high-dimensional feature

spaces, such as scRNA-seq data, where a comprehensive encapsulation of causal information relative to y is achievable. This ensures the reliability of causal inferences in complex biological datasets where not all variables may be observable.

Note S10. Staged training strategy

To effectively balance these different loss components, we employ a staged training strategy that focuses on different losses at various stages.

In the initial stage, approximately the first 10% of the epochs, the primary focus is on minimizing the ELBO loss $\mathcal{L}_{\text{ELBO}}$, specifically emphasizing the reconstruction losses for the causal and spurious VAEs ($\mathcal{L}_{\text{rec1}}$ and $\mathcal{L}_{\text{rec2}}$). This stage aims to embed the latent features z within the data manifold and ensure accurate data reconstruction. The loss for $\mathcal{L}_{\text{BCE}}$, $\mathcal{L}_{\text{causal}}$ and $\mathcal{L}_{\text{fidelity}}$ are not considered in this phase.
From approximately 10% to 40% of the epochs, the emphasis within the ELBO loss expands to include the KL divergence loss. This transition helps in regularizing the latent space by enforcing a prior distribution over the latent variables, which is crucial for a well-behaved latent space.

From approximately 40% to 70% of the epochs, the focus extends to include the binary cross-entropy (BCE) loss $\mathcal{L}_{\text{BCE}}$. This phase aims to enhance the accuracy of phenotype or state predictions by directly optimizing for the prediction task.

In the final stage, constituting approximately the final 30% of the epochs, the focus extends to include minimizing the causal-related losses, specifically the causal loss $\mathcal{L}_{\text{causal}}$ and the fidelity loss $\mathcal{L}_{\text{fidelity}}$. This phase ensures that the causal relationships are accurately captured and the representation of causal features is refined.

This staged approach ensures a balanced and comprehensive optimization process, enabling CauFinder to effectively distinguish and manipulate causal factors.

Note S11. CauFinder architecture

CauFinder employs a Dual Variational Autoencoder (DVAE) architecture, implemented using the PyTorch (version 1.13.0)[8] and Scanpy (version 1.9.1)[18] Python libraries. This architecture integrates several key components to facilitate causal modeling and network control, comprising a feature selection layer, an encoder, a decoder, and a classifier for binary cross-entropy (BCE) loss.

The feature selection layer is the first layer applied to the input data $=x$. It splits the input features into causal and spurious components using initial weights and a threshold. An optional attention mechanism, implemented through an attention network with two linear layers and LeakyReLU activations, refines the feature selection process.

The encoder projects the input vector to 128 dimensions, followed by BatchNorm1d batch normalization, ReLU non-linear activation, and Dropout regularization with a dropout rate of 0.1. It consists of two modules: one projects causal features x_1 into a latent space z^c with 2 dimensions (default), and the other projects spurious features x_2 into a latent space z^s with 8 dimensions. Both modules utilize multiple layers, batch normalization, ReLU activation, and the reparameterization trick for sampling.

The decoder mirrors the encoder architecture. It first applies a fully connected layer and ReLU activation to project the latent representation back to 128 dimensions, then projects this 128-dimensional layer into outputs matching the input vector size. The decoder also comprises two modules: one reconstructs x_{rec1} from z^c, and the other reconstructs x_{rec2} from z^s. The decoder employs a dropout rate of 0.0.

The classifier for binary cross-entropy (BCE) loss predicts the phenotype or state y. This classifier uses the latent representations z^c and z^s to perform binary classification, enhancing the accuracy of phenotype or state predictions. The classifier applies a fully connected layer to the concatenated latent vectors and outputs the predicted probabilities.

This DVAE architecture, combined with the feature selection layer and classifier, ensures that CauFinder effectively captures and manipulates causal and spurious factors, enabling precise guidance of phenotype transitions.

Note S12. Datasets and data preprocessing

Human and mouse lung adenosquamous carcinoma (LUAS) bulk sequencing datasets were obtained from the original studies[19]. These datasets provide comprehensive sequencing data for 93 human LUAS samples and for mouse samples at various stages post-Ad-Cre administration: atypical adenomatous hyperplasia at 4 weeks, LUAD at 6 and 7 weeks, and LUSC at 8, 9, and 10 weeks. To facilitate our analysis, we applied a logarithmic transformation to the datasets.

For single-cell sequencing data, the data from previous study[20] were utilized in our research, including scRNA-seq data with the Watermelon system, as well as processed cell information provided in the form of a meta-data matrix, which included critical information such as sequencing time, majority fate, and clone size. We preprocessed the data using Scanpy (v1.9.3) according to the methods provided in the original study. For each cell, we quantified the number of expressed genes and the proportion of transcripts from mitochondrial-encoded genes. Cells with fewer than 1,000 or more than 4,200 detected genes or a mitochondrial fraction greater than 0.1 were excluded from further analysis. Finally, the expression matrix was filtered to remove genes detected in fewer than three cells. This preprocessing resulted in a dataset containing 56,419 Watermelon-PC9 cells for the main analysis and an additional 16,477 cells from Watermelon models of EGFR-driven lung cancer (PC9) as an independent supplement, using a dispersion cutoff of 0.5, resulting in 1,296 highly variable genes. The preprocessed single-cell data were classified and labeled according to the provided cell information, resulting in several subsets used as input for CauFinder. Time-based groupings were classified according to known time labels, and cycling persister cells and non-cycling counterparts similarly classified based on given labels. For classifications not provided, such as cycling persister cells appearing on day 0, clonal barcodes were used to trace clonal lineages, enabling the tracking of cells' clonal origin and their proliferative and transcriptional states.

For drug resistance analysis, we categorized the dataset into two groups based on cell sensitivity to Osimertinib. Specifically, day0 cells with lineage barcodes that were detected in persister stages (days 3, 7, and 14) were labeled as "resistant-lineage," while others were labeled as "sensitive-lineage." This partition allowed for the specific identification of drivers associated with the emergence of resistance.

Spatial transcriptomics datasets for primary colorectal cancer (P1) and paired liver metastasis (LM1) were obtained from a recent study. We utilized the MCGAE algorithm to cluster the spatial transcriptomics data. For the P1 sample, clusters corresponding to tumor tissue and normal mucosa were identified. For the LM1 sample, clusters representing the tumor core, liver tissue, and the tumor-liver interface were annotated based on spatial location and marker gene expression.

Supplementary figures

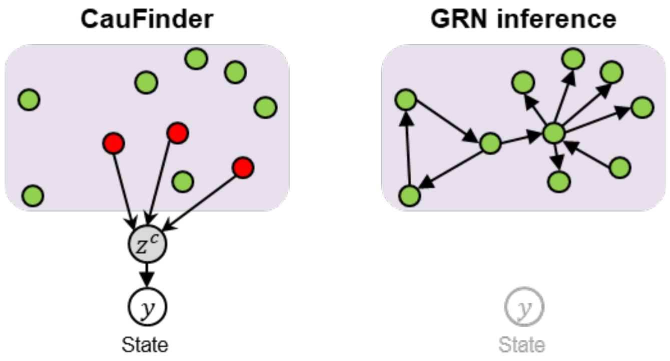

Figure S1: Scope Comparison: CauFinder vs. General GRN Inference.
Traditional Gene Regulatory Network (GRN) inference reconstructs gene-to-gene interactions, identifying the relationships from transcription factor (TF) to target gene (TG) without explicitly modeling their effects on a state transition. Instead of inferring direct regulatory edges between genes, CauFinder identifies the relationships from a set of causal genes to a state transition, i.e. identifies key causal drivers of a state transition by disentangling true causal effects from spurious correlations. It further integrates causal inference with network control to quantify their impact on the transition process, enabling more targeted interventions. The red dots in the figure indicate the set of the drivers for the state y, identified by CauFinder, while the green dots are other spurious genes to the state transition.

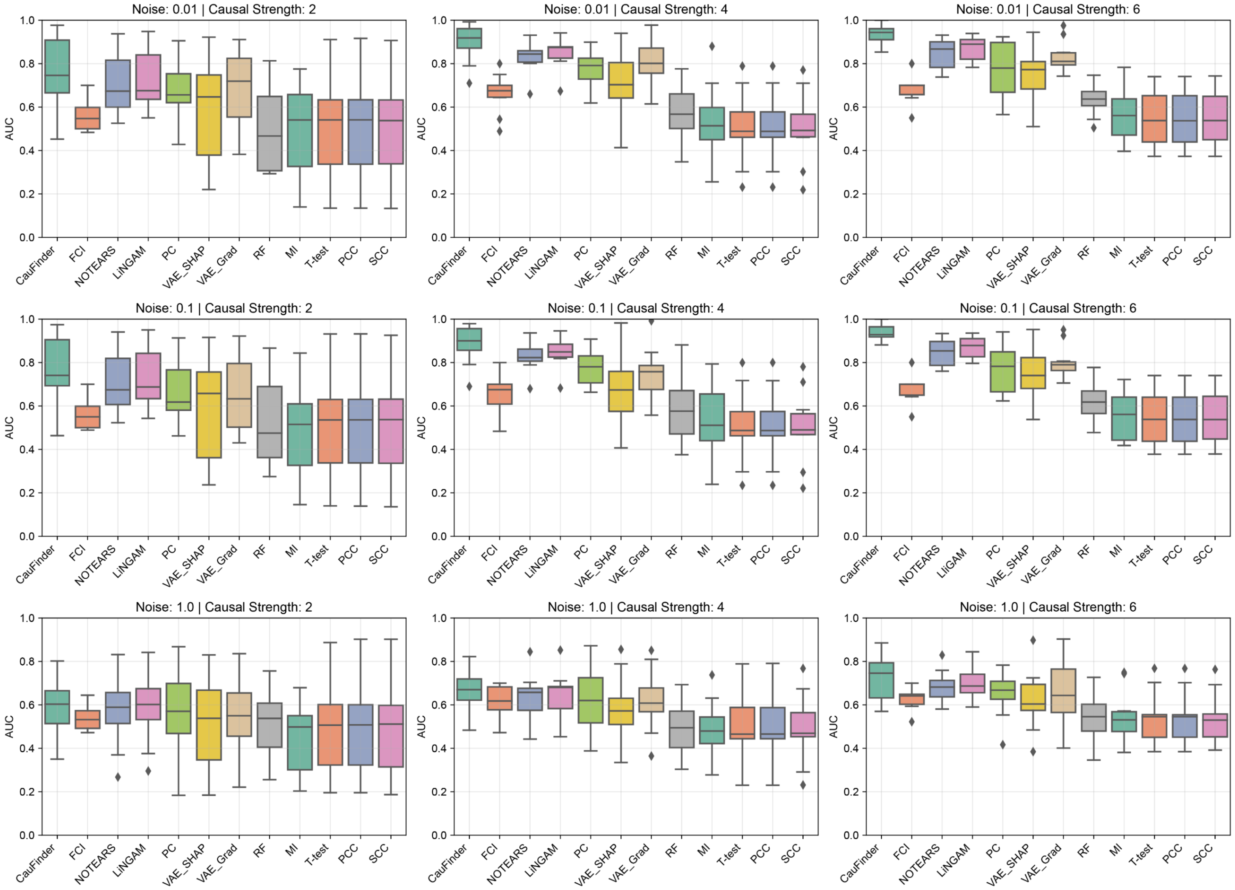

Figure S2. Comparison of AUC values for different methods across various causal strengths and noise levels based on linear synthetic simulation data.

The nine-panel box plot grid displays the AUC (Area Under the Curve) values for our model and other competing models under different simulated conditions. Each panel represents a specific combination of causal strength (0.2, 0.4, 0.6) and noise level (0.01, 0.1, 1.0). The methods compared include CauFinder, FCI, NOTEARS, LiNGAM, PC, VAE-SHAP, VAE-Grad, RF, MI, T-test, PCC, and SCC. The y-axis in each box plot represents the AUC values, illustrating the performance variability and robustness of each method under varying causal strengths and noise levels, based on linear simulated data.

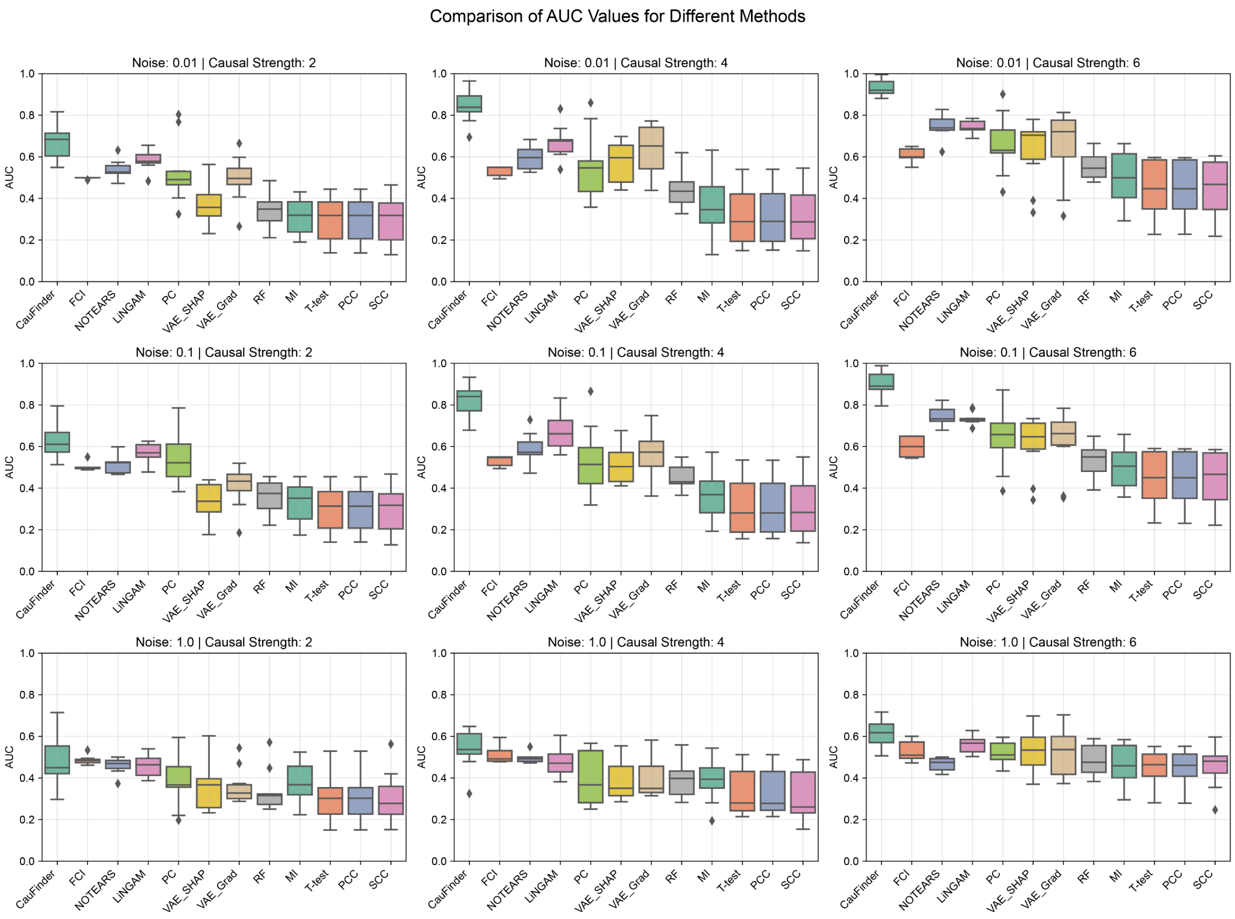

Figure S3. Comparison of AUC values for different methods across various causal strengths and noise levels based on nonlinear synthetic simulation data.

The nine-panel box plot grid displays the AUC (Area Under the Curve) values for our model and other competing models under different simulated conditions. Each panel represents a specific combination of causal strength (0.2, 0.4, 0.6) and noise level (0.01, 0.1, 1.0). The methods compared include CauFinder, FCI, NOTEARS, LiNGAM, PC, VAE-SHAP, VAE-Grad, RF, MI, T-test, PCC, and SCC. The y-axis in each box plot represents the AUC values, illustrating the performance variability and robustness of each method under varying causal strengths and noise levels, based on nonlinear simulated data.

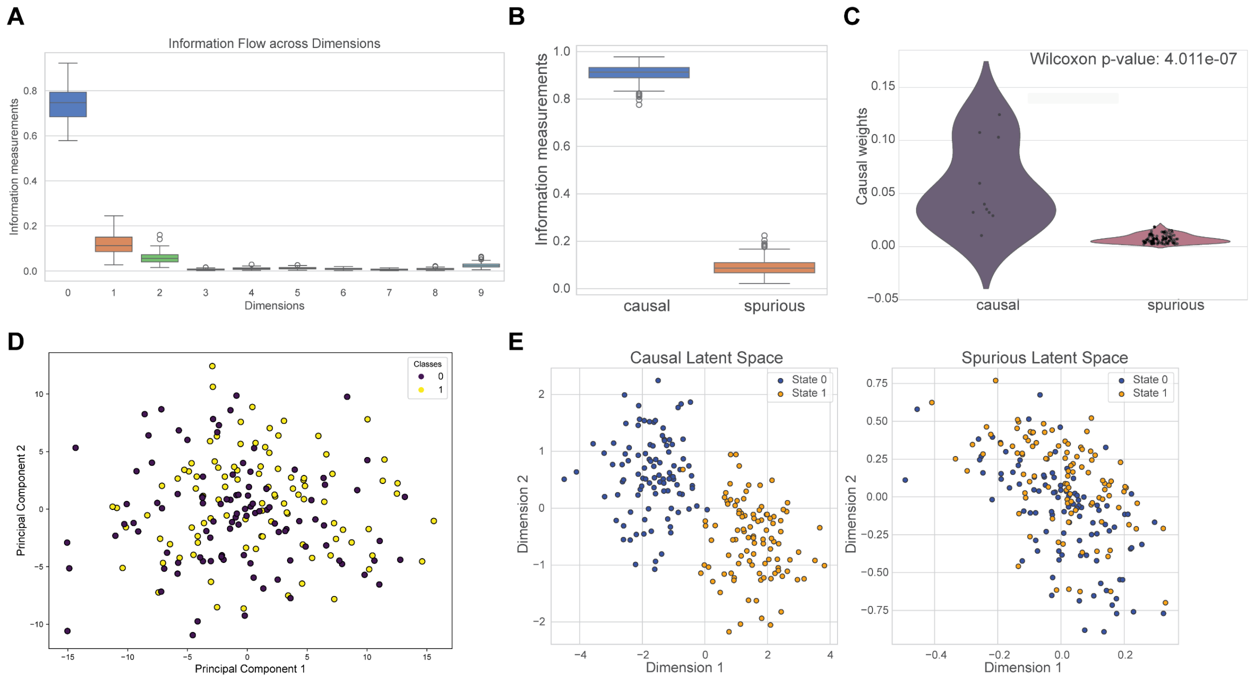

Figure S4. Causal decoupling performance of CauFinder on synthetic simulation data.

(A) Causal information flow across different dimensions of the latent space. The x-axis represents the dimensions, and the y-axis represents the causal information flow values. Each boxplot point represents a sample.

(B) Boxplot illustrating the distribution of information measurements for the causal dimensions (left) and spurious dimensions (right) in the model's latent space. The y-axis represents the information measurements.

(C) Violin plot displays the distribution of feature weights assigned by the model to causal features (left) and spurious features (right), with violin width indicating feature density.

(D) Principal component analysis plot of the simulated data. The x-axis and y-axis represent the first two principal components, respectively. Colours indicate the two classes (Class 0 and Class 1).

(E) Scatter plots compare the sample distributions in the model's latent space based on causal dimensions (left) and spurious dimensions (right). Each point represents a sample, colored by class (Class 0 in blue and Class 1 in orange).

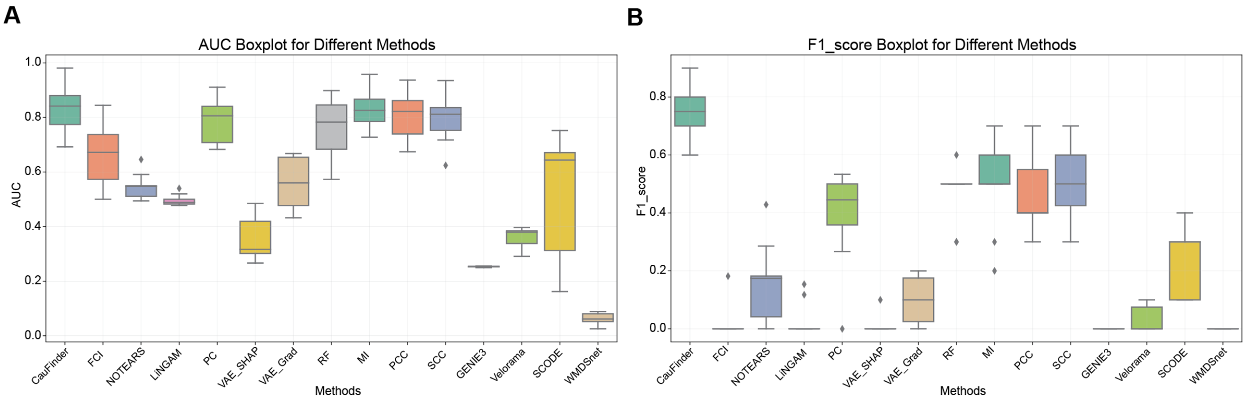

Figure S5. Performance comparison of CauFinder and other methods on perturbation-based simulation data.

Boxplots showing the AUC values and F1-scores for CauFinder and other competing methods on the perturbation-based simulation dataset. The methods compared include CauFinder, FCI, NOTEARS, LiNGAM, PC, VAE_SHAP, VAE_Grad, RF (Random Forest), MI (Mutual Information), PCC (Pearson Correlation Coefficient), SCC (Spearman Correlation Coefficient), GENIE3, Velorama, SCODE, and WMDSnet. The central line in each box represents the median, the box edges represent the 25th and 75th percentiles, and the whiskers extend to the most extreme data points not considered outliers.

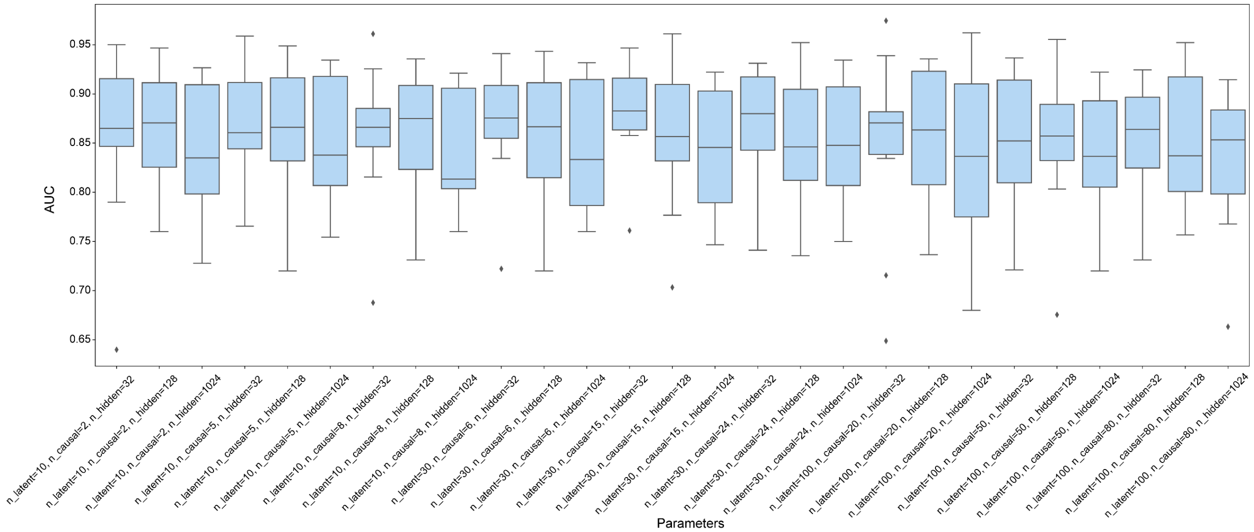

Figure S6. Performance (AUC) of CauFinder across different hyperparameter settings.
AUC scores obtained from multiple runs for each combination of hyperparameters. The hyperparameters explored are: n_latent (number of latent dimensions: 10, 30, 100), n_causal_pct (percentage of latent dimensions used for causal factors: 0.2, 0.5, 0.8), and n_hidden (number of hidden units in the neural networks: 32, 128, 1024). Each boxplot is labeled with the specific hyperparameter combination used (e.g., "n_latent=10, n_causal=2, n_hidden=32"). The central line in each box represents the median, box edges represent the 25th and 75th percentiles, whiskers extend to the most extreme non-outlier data points, and outliers are plotted individually.

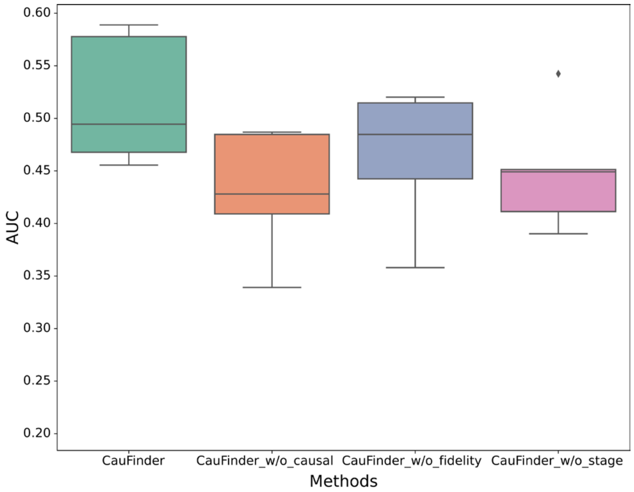

Figure S7. Performance comparison (AUC) of CauFinder and its variants in an ablation study. Each boxplot shows the distribution of AUC scores obtained from multiple independent runs. The variants include: CauFinder (full model, green), CauFinder without the causal loss component ($\mathcal{L}_{\text{causal}}$, orange), CauFinder without the fidelity loss component ($\mathcal{L}_{\text{fidelity}}$, blue), and CauFinder without staged training (pink). The central line in each box represents the median, the box edges represent the 25th and 75th percentiles, and the whiskers extend to the most extreme data points not considered outliers. Outliers are plotted individually as points.

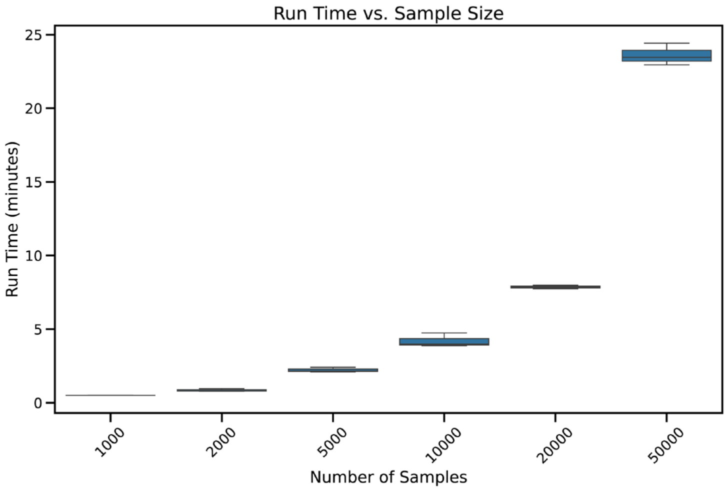

Figure S8. Runtime analysis of CauFinder: Run time (minutes) versus the number of samples. This boxplot illustrates the computational run time (in minutes) of CauFinder as a function of the sample size. The x-axis represents the number of samples, ranging from 1,000 to 50,000, while the y-axis shows the corresponding run time in minutes. Each boxplot displays the variation in run time across multiple runs for a given sample size. The central line in each box represents the median, the box edges represent the 25th and 75th percentiles, and the whiskers extend to the most extreme non-outlier values. The results demonstrate the scalability of CauFinder, showing an approximately linear increase in run time with respect to the number of input samples. Experiments were conducted on a Windows workstation equipped with an Intel Core i9-12900KF CPU (3.19 GHz, 128 GB RAM) and an NVIDIA GeForce RTX 3070 GPU (8 GB VRAM).

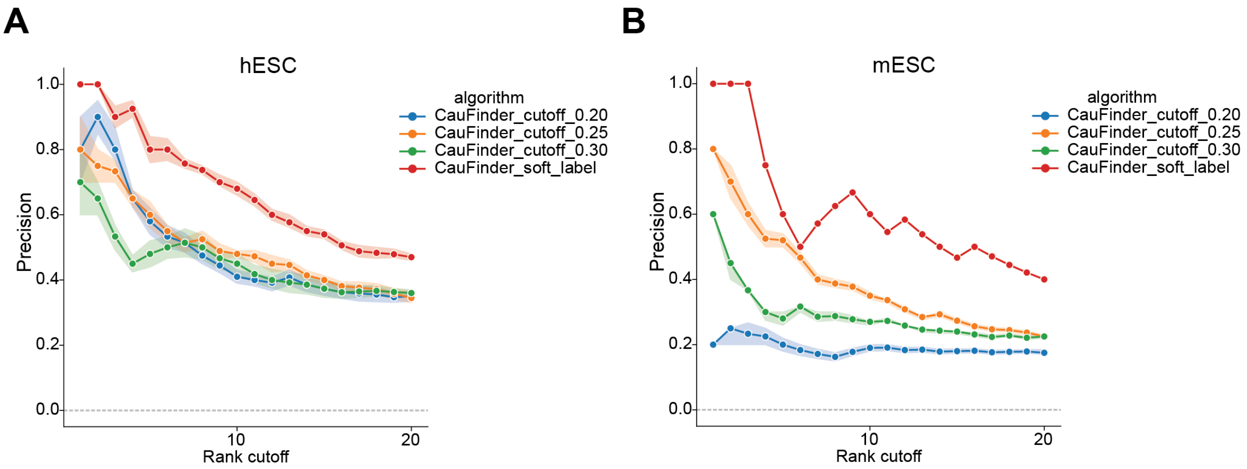

Figure S9. Impact of early/late stage demarcation criteria on CauFinder's performance in embryonic stem cell (ESC) datasets.

(A) Human embryonic stem cell (hESC) dataset and (B) Mouse embryonic stem cell (mESC) dataset: Precision of top-ranked gene predictions under different early/late stage demarcation strategies. Three fixed cutoff-based approaches (blue: 20%, orange: 25%, green: 30%) and a soft-labeling approach (red) were evaluated. Precision values were computed based on a reference gene set associated with stem cell fate and differentiation. The results illustrate variations in precision across different thresholding choices, highlighting the influence of early/late stage definitions on performance evaluation.

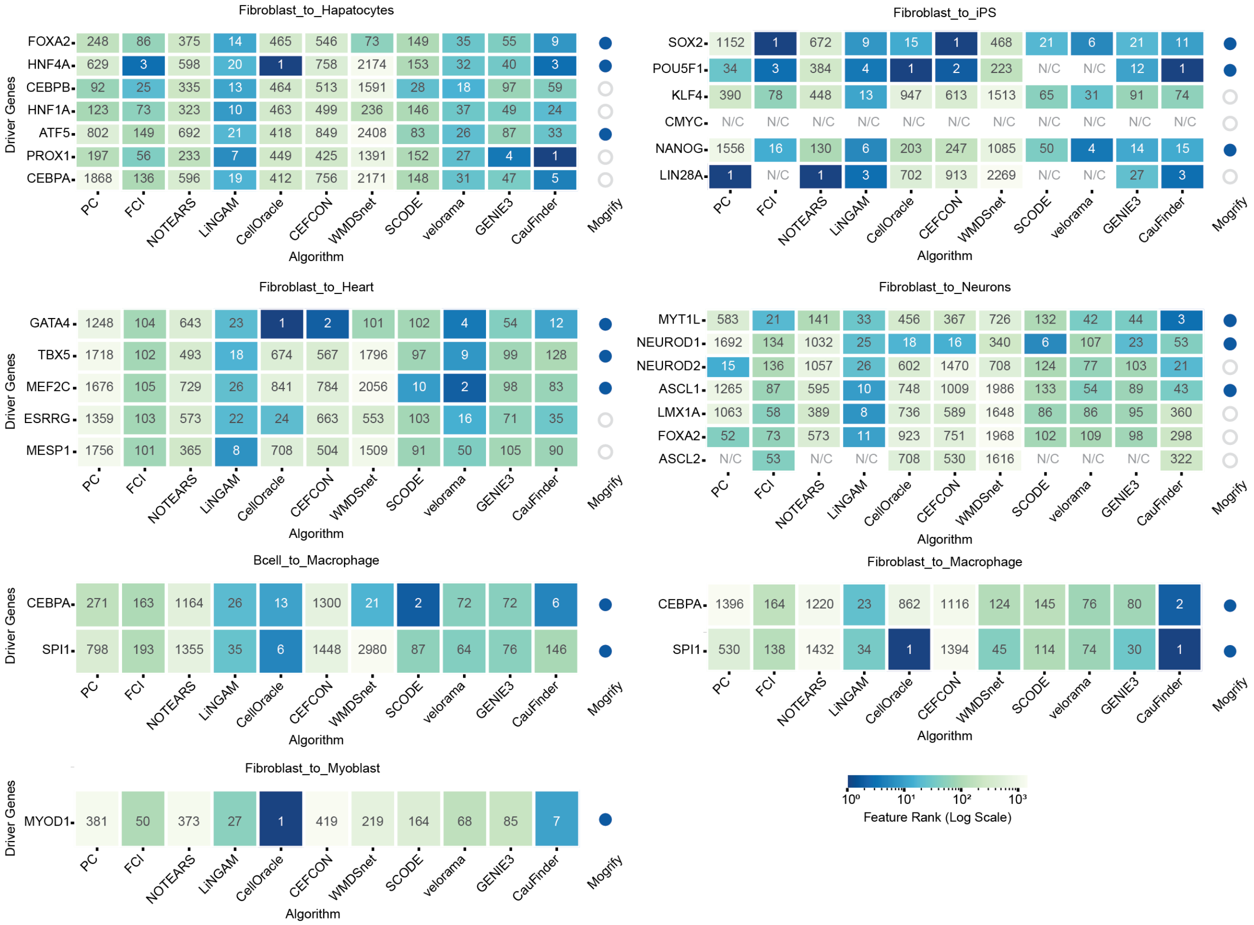

Figure S10. Benchmark evaluation of CauFinder on literature-curated known cell conversion systems.

Heatmaps showing the ranking positions of experimentally validated regulators across seven classical cell fate conversion settings, including fibroblast-to-neuron, fibroblast-to-myoblast, fibroblast-to-hepatocyte, fibroblast-to-iPSC, fibroblast-to-macrophage, fibroblast-to-cardiomyocyte, and B cell-to-macrophage transitions. Rows represent literature-supported regulatory factors curated from the original experimental studies, and columns represent different computational methods. Values indicate the ranking position of each regulator among candidate genes, with lower values corresponding to higher prioritization. N/C indicates regulators not covered in the corresponding candidate gene list. Filled and open circles in the Mogrify column indicate regulators reported and not reported by Mogrify, respectively.

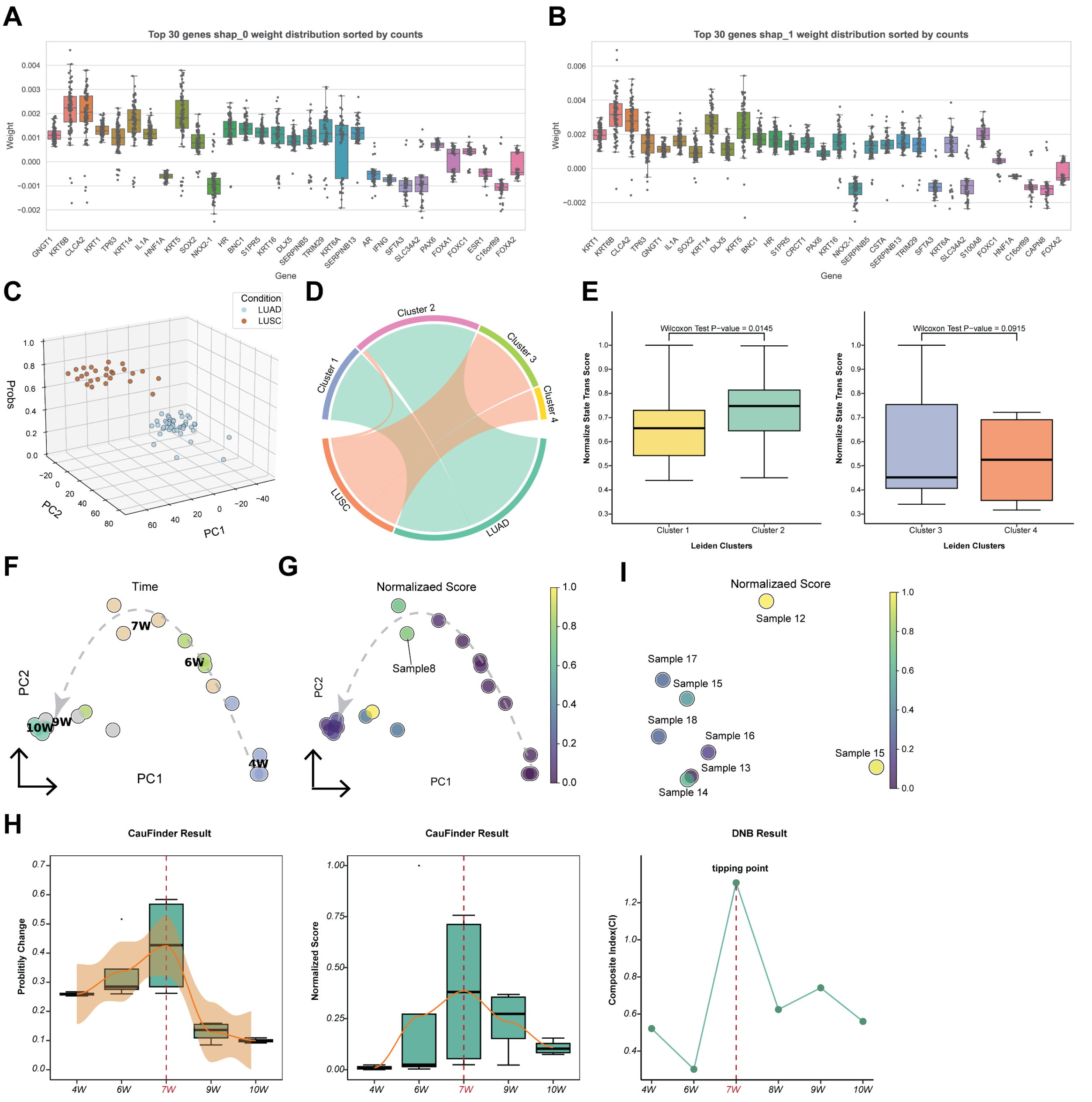

Figure S11. Identifying causal drivers igniting the transdifferentiation between lung adenocarcinoma and squamous cell carcinoma.

(A-B) Boxplots with individual data points showing the distribution of causal weight scores for the top 30 genes. These genes were selected based on their frequency of occurrence across 100 runs. The horizontal axis lists the genes, and the vertical axis represents the causal weight scores derived from the CauFinder model. (A), Transition from lung adenocarcinoma (LUAD) to lung squamous cell carcinoma (LUSC). (B), Reverse transition from LUSC to LUAD. (C) 3D visualization of all LUAD and LUSC samples using principal component analysis (PCA). The x and y axes represent PC1 and PC2, respectively, while the z-axis shows the state score of each sample. (D) Chord plot shows the clustering of samples using the Leiden algorithm with a resolution of 0.25. (E) Boxplot shows the normalized state transition scores calculated by CauFinder during the simulated state transitions for LUAD samples (Cluster 1 and Cluster 2) and LUSC samples (Cluster 3 and Cluster 4), grouped by cluster. The P value is indicated at the top (based on the Wilcoxon rank-sum test). (F-G) PCA visualization of RNA-seq data revealing the trajectory of phenotypic transitions

from LUAD to LUSC. Each node represents one sample, with the time points (weeks) of sampling shown (F), and state transition score of samples on the same PCA embedding (G). (H) Boxplots showing model-derived state probability transitions (left panel) and controllability scores (middle panel) at different time points. The line graph (right panel) displays the composite index (CI) for quantifying the tipping point of system state, based on previous research. The 7-week mark (7W) is highlighted as the tipping point. (I) State transition scores of samples at 9 and 10 weeks on the PCA embedding, illustrating the transition from LUSC to LUAD.

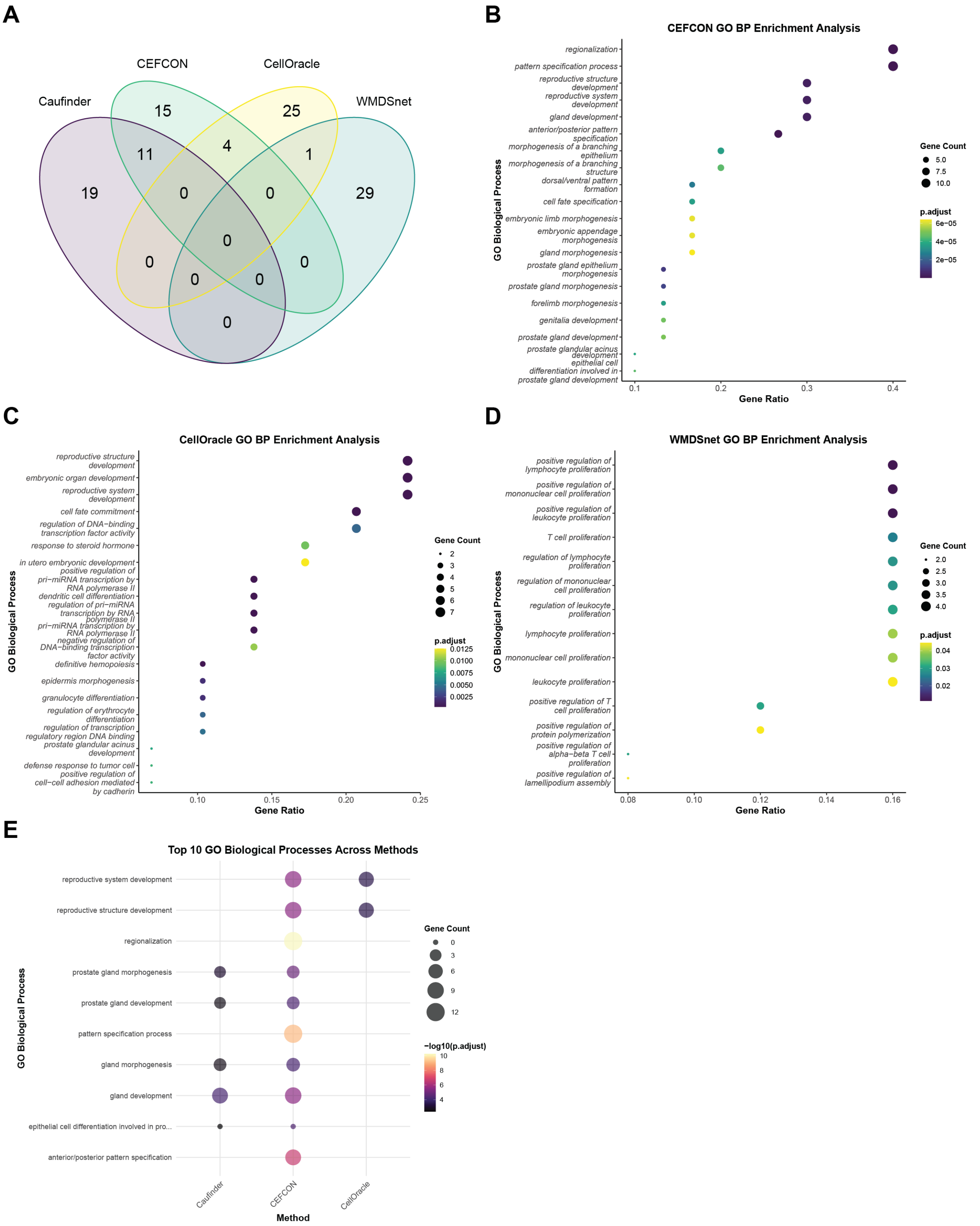

Figure S12. Comparative analysis of computational methods in lung adenocarcinoma and squamous cell carcinoma.

(A) Venn diagram illustrating overlap of driver genes identified by four computational methods (CEFCON, CellOracle, CauFinder, WMDSnet). Numerical labels indicate unique and shared gene counts across methods. (B–D) GO biological process (BP) enrichment analysis for (C) CEFCON, (D) CellOracle, and (E)

WMDSnet, ranked by adjusted p-value (FDR < 0.05). Dot sizes represent gene counts; color scales denote statistical significance. (E) Consolidated visualization of top 10 enriched pathways across all methods. Bar heights reflect gene ratios (enriched genes/total background genes), with color coding corresponding to methods.

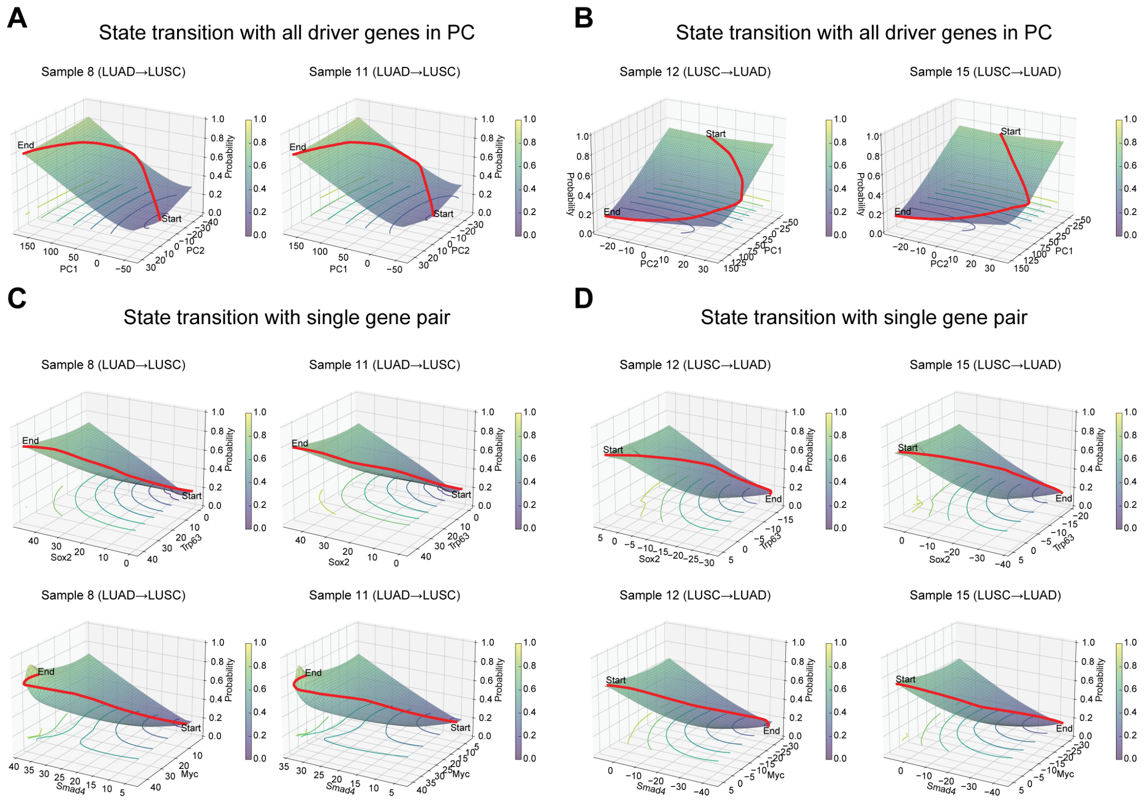

Figure S13. Computational simulation of the state transitions between LUAD and LUSC, controlled by drivers identified through CauFinder.

The figure is organized to showcase both the natural transition from LUAD to LUSC (A, C) and potential reversed transition from LUSC to LUAD (B, D). (A) and (B) represent the transitions controlled by all drivers identified by CauFinder after PCA dimensionality reduction, offering a comprehensive view of how these drivers influence the state changes. In contrast, panels c and d focus on transitions governed by specific gene pairs. The upper panels (C) and (D) highlight gene pairs that have been experimentally validated in previous studies, while the lower panels showcase our newly identified gene pair, *Myc* and *Smad4*. Each panel plots gene expression against a state probability score as defined by CauFinder, where values approaching 0 indicate proximity to LUAD, and values nearing 1 denote proximity to LUSC.

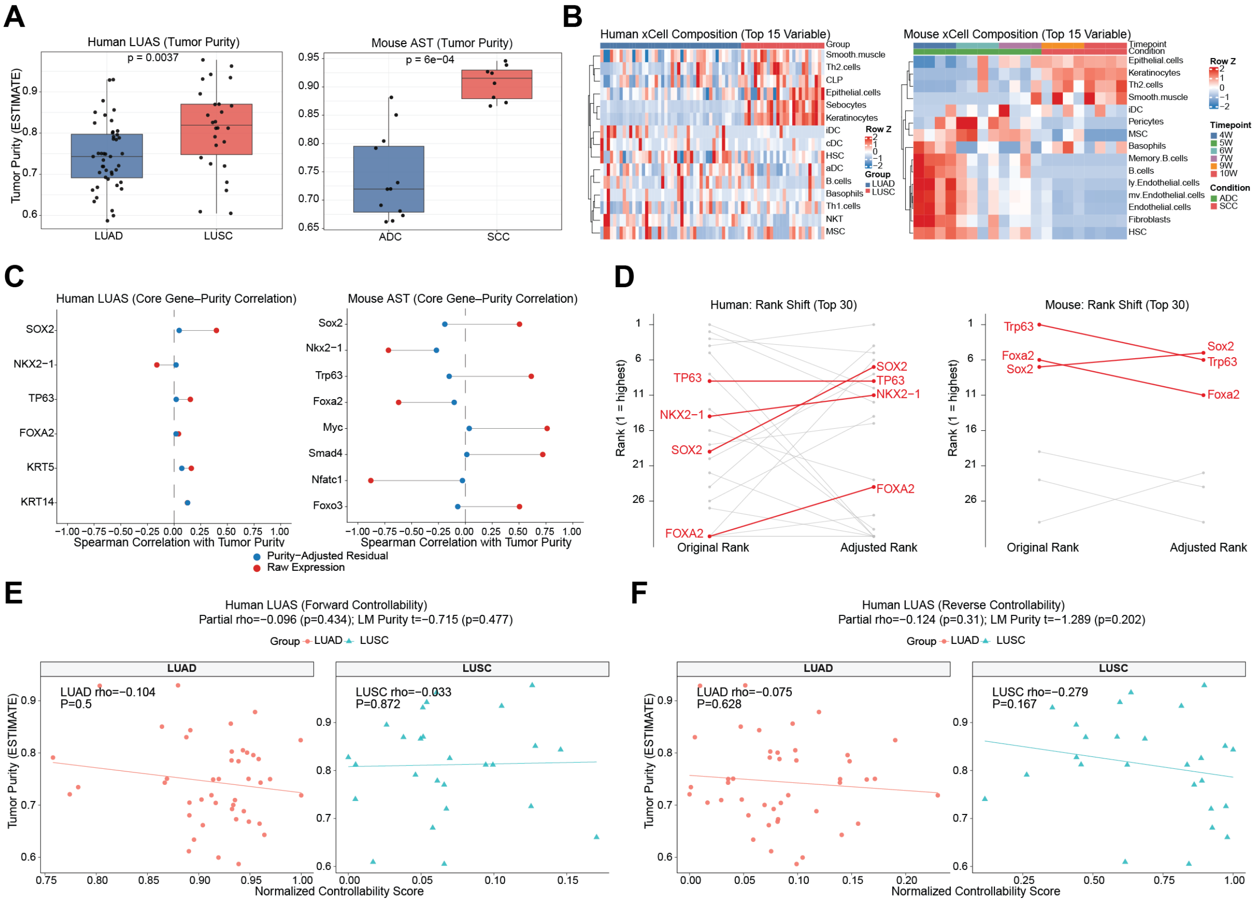

Figure S14. Deconvolution-based assessment of compositional confounding and robustness of CauFinder-prioritized regulators after purity/TME adjustment.

(A) Estimated tumor purity in the bulk datasets. Left, human LUAS samples stratified as LUAD-like and LUSC-like; right, mouse AST samples stratified as ADC and SCC. Tumor purity was inferred using ESTIMATE. (B) Heatmaps of major tumor microenvironment components inferred by xCell. Left, human LUAS; right, mouse AST. Displayed are the most variable cell-type scores, highlighting subtype/stage-associated differences in stromal and immune composition. (C) Correlation of representative core regulators with tumor purity before and after compositional adjustment. Left, human LUAS; right, mouse AST. Spearman correlations with tumor purity are shown for raw expression and purity/TME-adjusted residual expression. (D) Rank-shift plots comparing CauFinder gene rankings before and after tumor purity and microenvironmental adjustment in the human LUAS cohort (left) and the mouse AST model (right). Key regulators, including *TP63, SOX2, NKX2-1,* and *FOXA2*, remained among the top-ranked candidates after adjustment. (E, F) Association between tumor purity and human sample-level controllability after accounting for LUAD/LUSC subtype structure. (E) Forward controllability (LUAD→LUSC). (F) Reverse controllability (LUSC→LUAD). Within-group Spearman correlations are shown separately for LUAD-like and LUSC-like samples, together with the group-adjusted partial correlation and linear-model statistics.

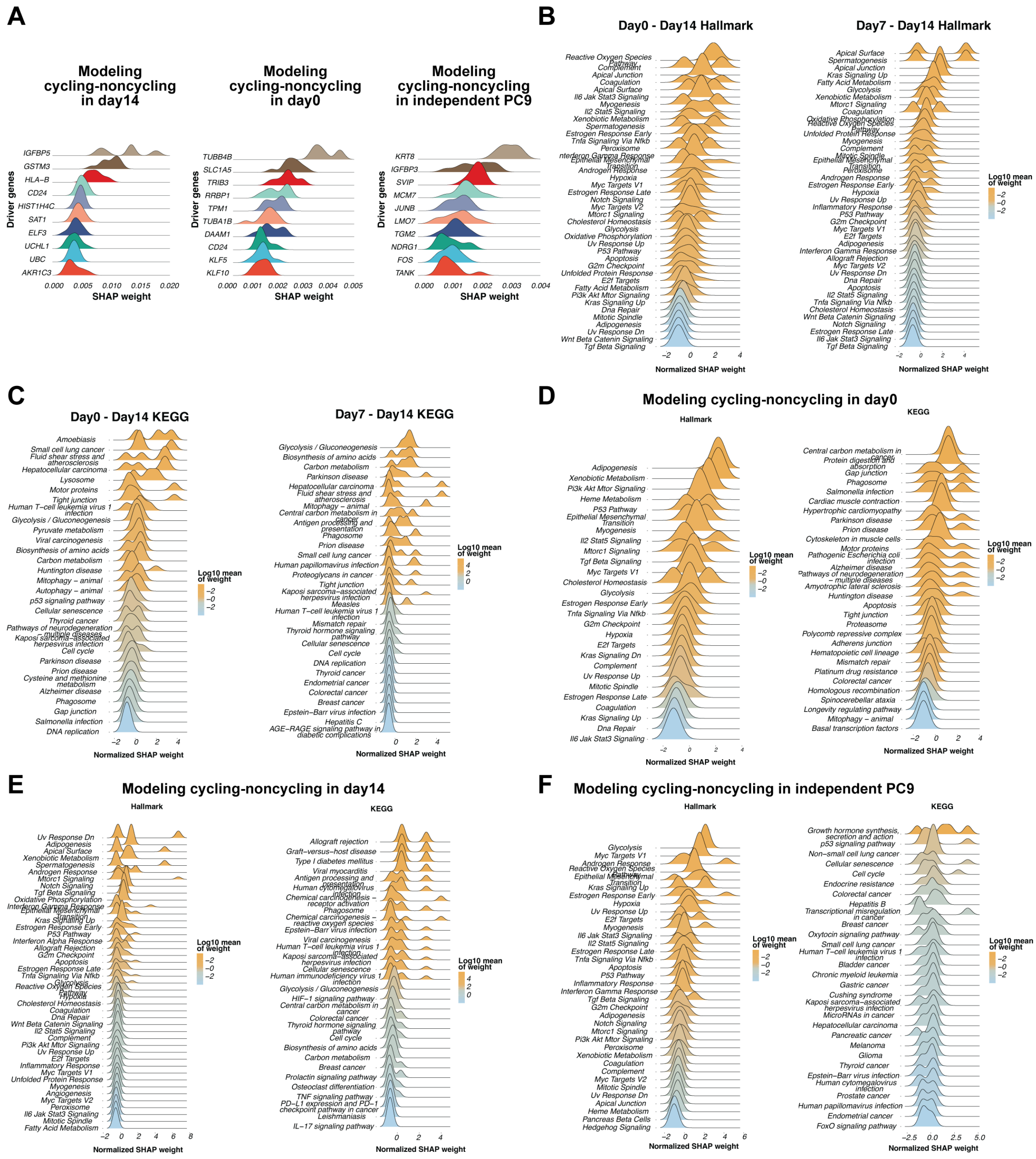

Figure S15. Causal genes identified by CauFinder and enrichment analysis results in cycling persister cells arising.
(A) The ridge plot shows the weights of the top 10 drivers predicted by CauFinder across ten calculations for the groups: day 14 (left), day 0 (middle), and EGFR-driven lung cancer (PC9) as an independent supplement (right). Persister cycling cells and non-cycling counterparts are labeled as 1 and 0, respectively. (B-C) Weight distribution of all causal drivers included in the HALLMARK pathways (B) and KEGG pathways (C) in day0 -day14 paired input CauFinder model (left) and day7-day14 paired input CauFinder model (right), ranked by the median weight of causal drivers within each pathway. All weights are log-transformed. (D-F) Weight distribution of all causal drivers included in the KEGG pathways and HALLMARK pathways in three different cycling-noncycling pairs: day0 (D), day14 (E), additional PC9 cell line (F).

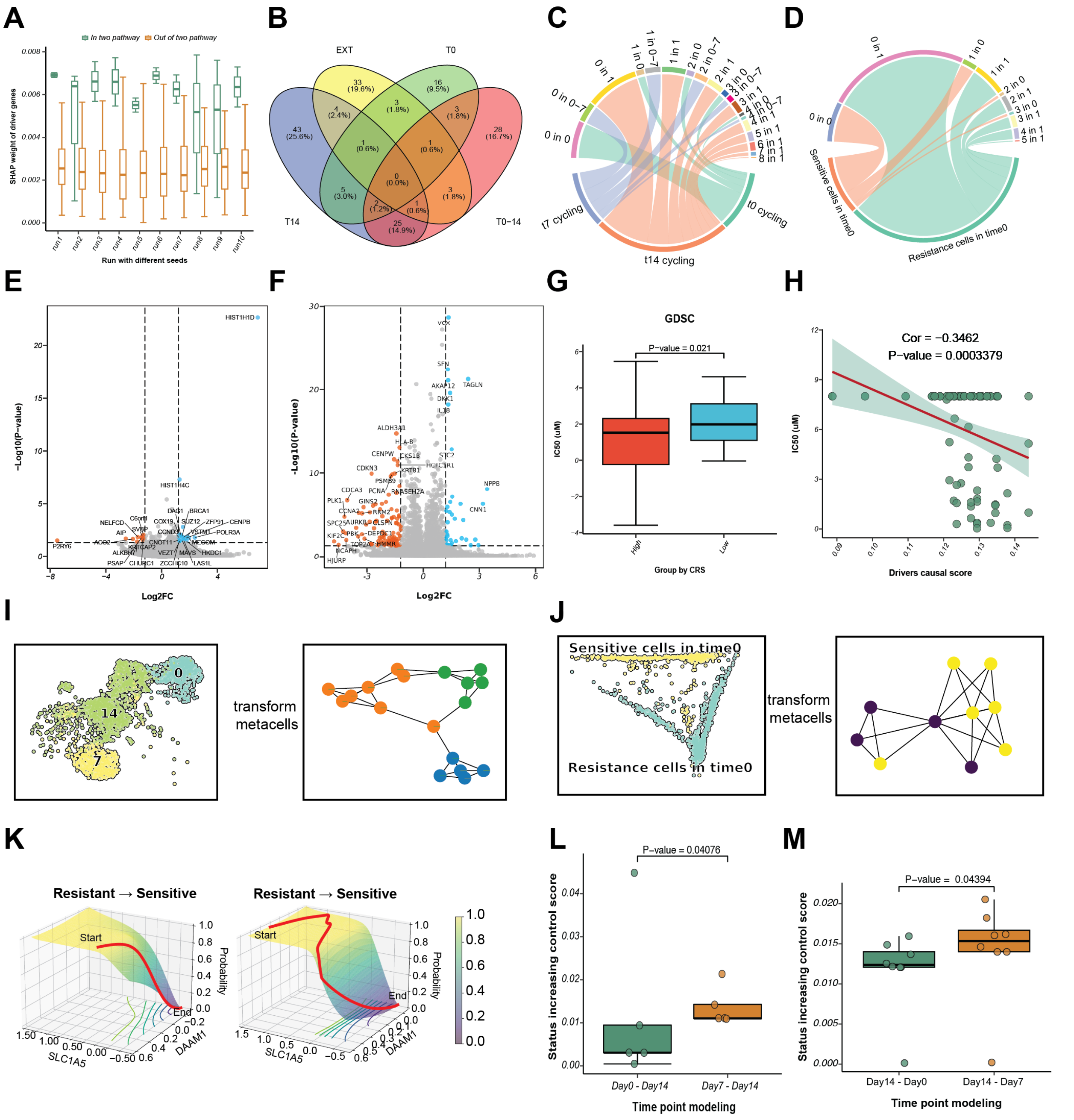

Figure S16. Downstream analysis of PC9 drug response single-cell datasets based on causal genes identified by CauFinder.

(A) Stability of causal driver weights across ten runs with different random seeds using fixed inputs (day0–day14 paired) and parameters. Drivers are grouped based on their involvement in ROS and FAM pathways. (B) The Venn plot illustrates the intersection of all drivers predicted by CauFinder, using pairs labeled as 1 for persister cycling cells and 0 for non-cycling counterparts as input. (C–D) Chord diagrams illustrating the correspondence between original cells and merged pseudo-cells used as inputs for state transition modeling. (C) Mapping based on sampling time points of persister cycling cells. (D) Mapping based on drug response states (sensitive versus resistant). (E–F) Volcano plots showing differential gene expression between pseudo-cells with extreme state transition scores and other pseudo-cells. (E) Comparison for day0 cells using pseudo-cells with highest transition scores. (F) Comparison for day14 cells using pseudo-cells with lowest transition scores. Blue dots indicate upregulated genes and red dots indicate downregulated

genes. (G) Boxplots showing the distribution of osimertinib IC_{50} values in GDSC cell lines across two groups categorized by the median causal driver scores. (H) Correlation between causal driver scores (x axis) and IC_{50} (y axis) in CCLE cell lines. (I–J) Construction of pseudo-cells and their structural relationships. (I) UMAP visualization of time-course data showing transformation of original cells into pseudo-cells. (J) UMAP visualization of drug-response data and corresponding pseudo-cell network representation. Edges in the pseudo-cell network are inferred using partition-based graph abstraction (PAGA). (K) Computational simulation of state transitions for different samples from the drug response pairs regulated by *DAAM1* and *SLC1A5*. The X-axis and Y-axis represent the expression levels of *DAAM1* and *SLC1A5*, respectively, while the Z-axis indicates the state probability, where values closer to 0 represent the defined starting point and values closer to 1 represent the defined endpoint. (L–M) Pseudo-cell path controllability scores under forward and reverse transition settings. (L) Forward transitions toward the day14 state. (M) Reverse transitions toward earlier states (day0 or day7). Statistical significance was assessed using the Kolmogorov–Smirnov test.

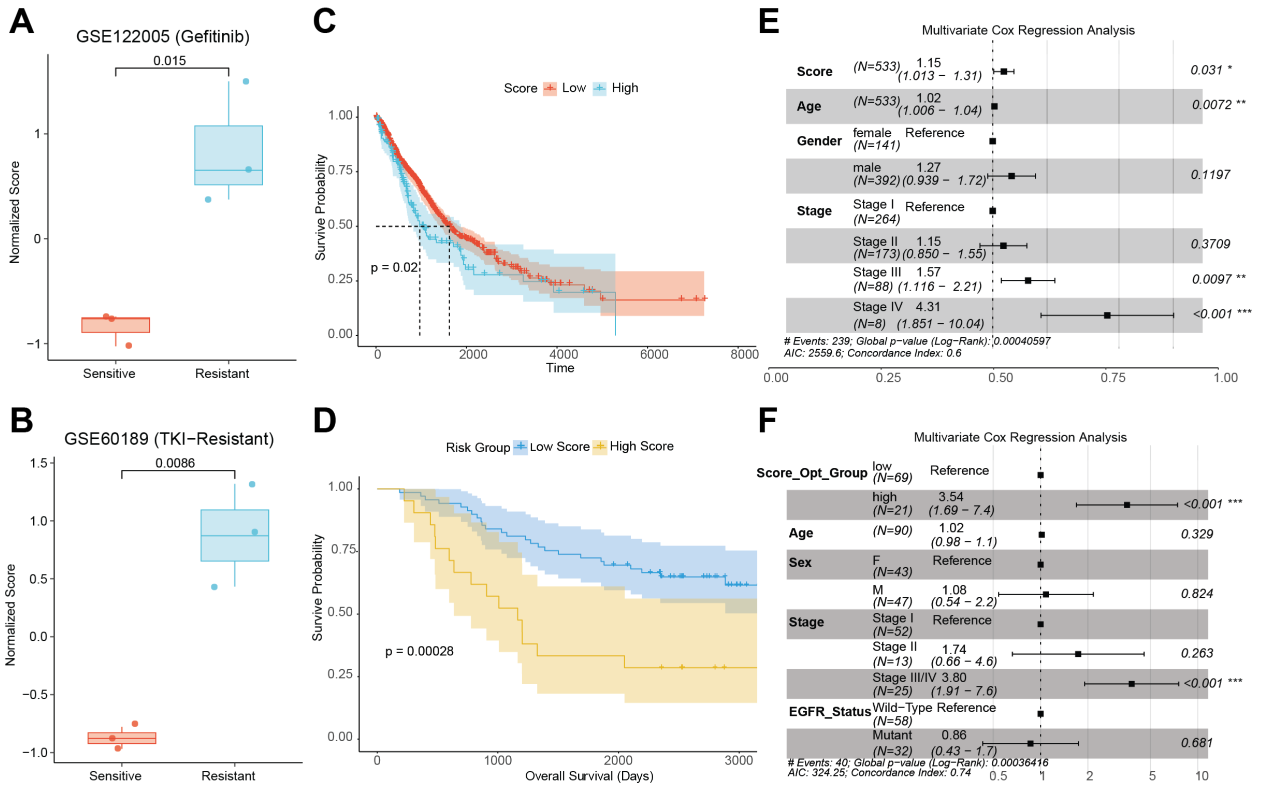

Figure S17. Validation of CRS in TKI resistance models and supportive clinical associations. (A–B) CRS in independent paired cell-line models of TKI sensitivity and acquired resistance. In GSE122005 (A), CRS was compared between gefitinib-sensitive parental cells and acquired gefitinib-resistant derivatives. In GSE60189 (B), CRS was compared between PC9 cells and gefitinib-resistant PC9/gef cells. P values are shown above the boxplots. (C–D) Kaplan–Meier survival curves of patients stratified by CRS. (C) Overall survival analysis in the combined TCGA lung cancer cohort. (D) Overall survival analysis in the independent GSE11969 cohort. P values were calculated using the log-rank test. (E–F) Multivariate Cox regression analysis of CRS and clinical covariates. (E) Cox regression in the TCGA LUSC subgroup. (F) Cox regression in the independent GSE11969 cohort. Hazard ratios and 95% confidence intervals are shown. Covariates included age, sex/gender, tumor stage, and EGFR mutation status where available.

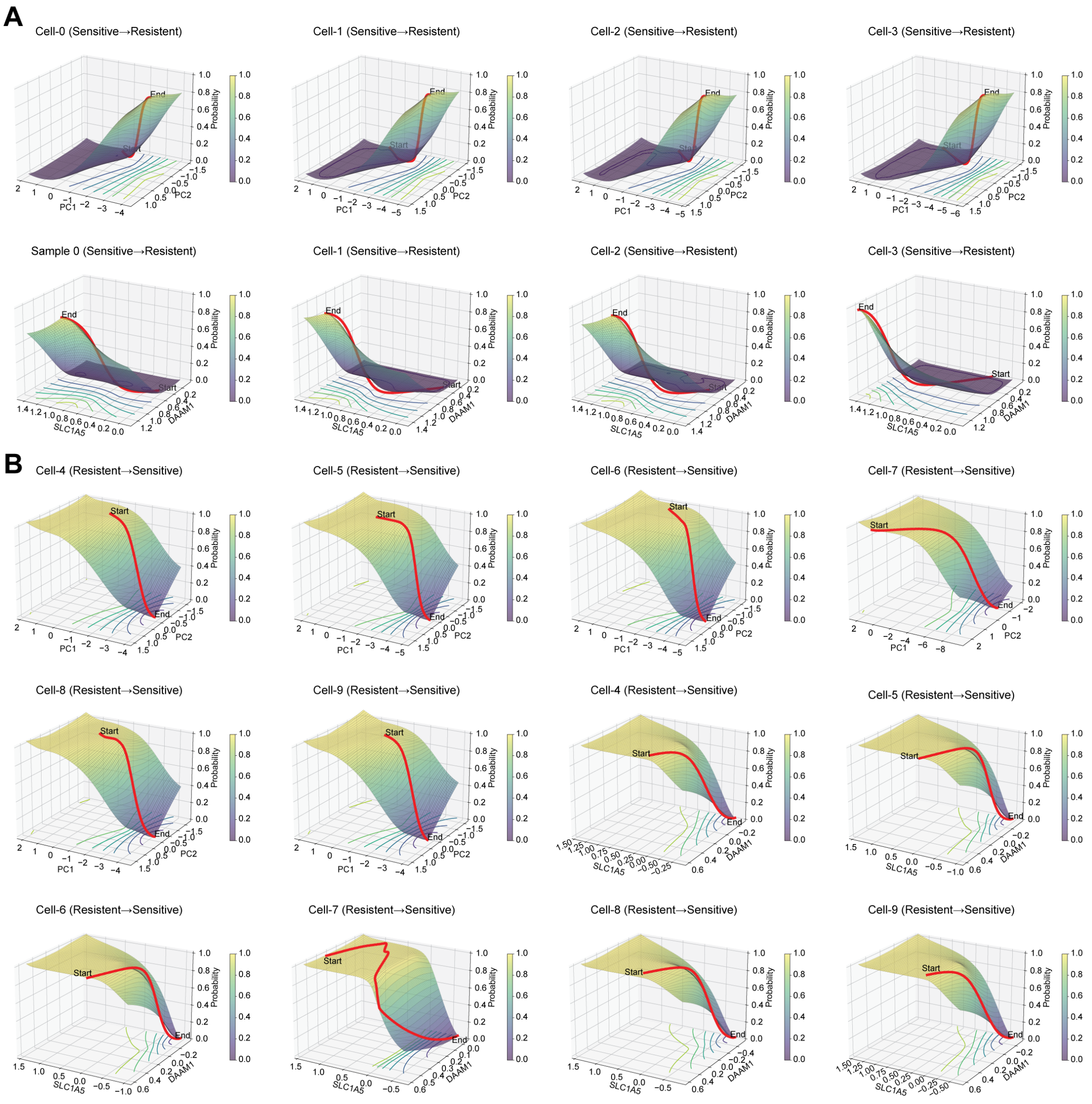

Figure S18. Computational simulation of state transition via control of causal genes in pseudo-cells.

(A) Simulation of state transition from drug-sensitive to resistant cells, using either all causal drivers (shown by PCA) or a combination of single biologically significant features (DAAM1 and SLC1A5). The upper panel shows the transition trajectory using PCA, while the lower panel focuses on the specific features. (B) Simulation of reverse state transition from drug-resistant to sensitive cells, using the same methods as in (A). The upper panel uses PCA, and the lower panel highlights the single significant features.

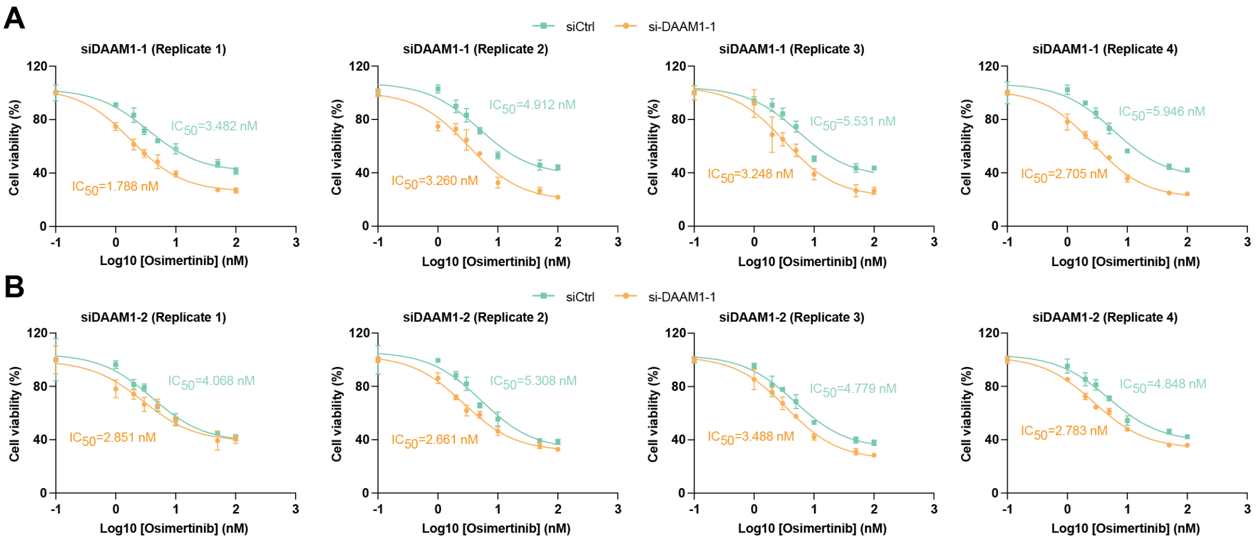

Figure S19. Independent replicate osimertinib dose-response curves following *DAAM1* knockdown in PC9 cells.

(A) Osimertinib dose-response curves from four independent biological replicates following siDAAM1-1 knockdown.(B) Osimertinib dose-response curves from four independent biological replicates following siDAAM1-2 knockdown.PC9 cells were transfected with non-targeting control siRNA or the indicated *DAAM1*-targeting siRNA and treated with a range of osimertinib concentrations for 72 h. Cell viability was measured using the CCK-8 assay, and dose-response curves were fitted to estimate IC_{50} values.

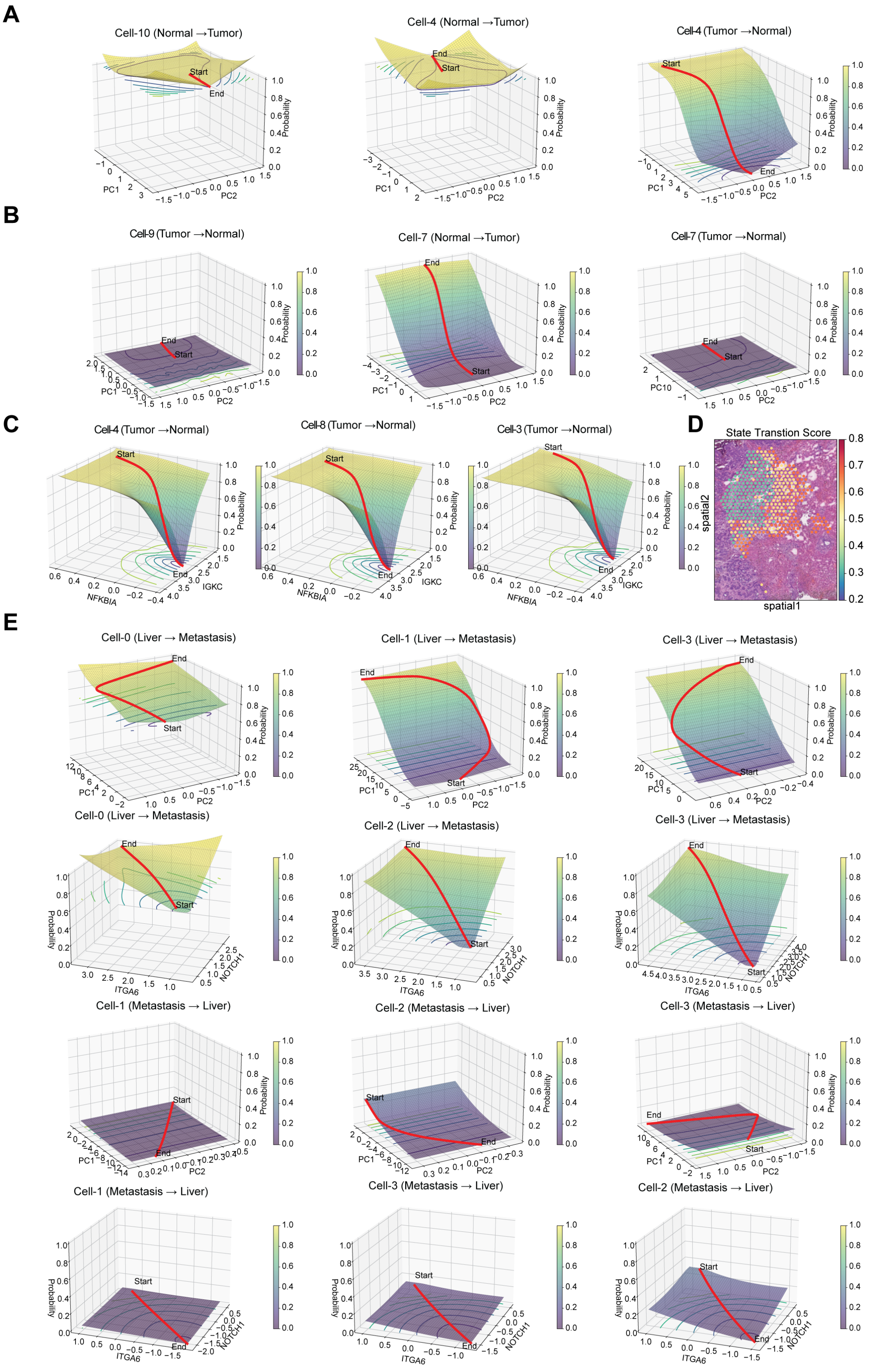
A
Cell-10 (Normal →Tumor)
Cell-4 (Normal →Tumor)
Cell-4 (Tumor →Normal)
B
Cell-9 (Tumor →Normal)
Cell-7 (Normal →Tumor)
Cell-7 (Tumor →Normal)
C
Cell-4 (Tumor →Normal)
Cell-8 (Tumor →Normal)
Cell-3 (Tumor →Normal)
D
State Transtion Score
spatial1
spatial2
E
Cell-0 (Liver → Metastasis)
Cell-1 (Liver → Metastasis)
Cell-3 (Liver → Metastasis)
Cell-0 (Liver → Metastasis)
Cell-2 (Liver → Metastasis)
Cell-3 (Liver → Metastasis)
Cell-1 (Metastasis → Liver)
Cell-2 (Metastasis → Liver)
Cell-3 (Metastasis → Liver)
Cell-1 (Metastasis → Liver)
Cell-3 (Metastasis → Liver)
Cell-2 (Metastasis → Liver)
Start
End
Probability
PC1
PC2
PC10
NFKBIA
IGKC
ITGA6
NOTCH1

Figure S20. Computational simulation of state transition of pseudo-cells between tumor and adjacent normal tissues, and between metastatic cancer and nearby liver tissues.
(A-B) Computational simulation of decreasing state transition from tumor to normal via control of causal genes for pseudo-cells from P1 tissue, based on either all drivers (shown by PCA, A) or a combination of single biologically significant features (NFKBIA and IGKC, B). (C) Computational simulation of decreasing state transition from tumor to normal via control of causal genes for cell-18 based on all drivers (shown by PCA). (D) Scatter plot in spatial coordinates showing the state transition score based on pseudo-cells at the tumor-liver tissue interface. (E) Computational simulation of increasing state transition via other pseudo-cells not mentioned in (Figure 5O).

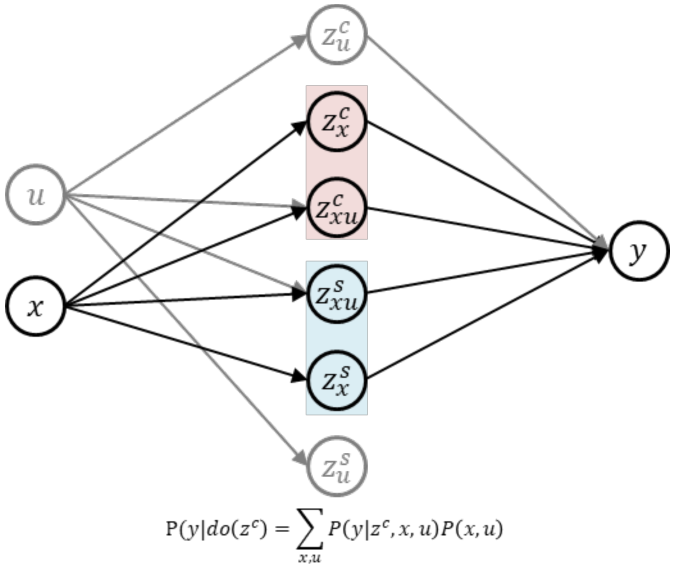

Figure S21. Structural causal model with unobserved variables.
In the causal graph, $\boldsymbol{x}$ represents observed variables, and $\boldsymbol{u}$ represents unobserved variables, which are further decomposed into causal and spurious components within the latent space. The unified causal latent spaces, $\boldsymbol{z}^c = \{\boldsymbol{z}_{xu}^c, \boldsymbol{z}_x^c\}$, consist of $\boldsymbol{z}_{xu}^c$, influenced by both $\boldsymbol{x}$ and $\boldsymbol{u}$, and $\boldsymbol{z}_x^c$, influenced solely by $\boldsymbol{x}$. Similarly, the unified spurious latent spaces, $\boldsymbol{z}^s = \{\boldsymbol{z}_{xu}^s, \boldsymbol{z}_x^s\}$, include $\boldsymbol{z}_{xu}^s$, affected by both $\boldsymbol{x}$ and $\boldsymbol{u}$, and $\boldsymbol{z}_x^s$, affected only by $\boldsymbol{x}$. The complete set of latent variables, $\boldsymbol{z} = \{\boldsymbol{z}^c, \boldsymbol{z}^s, \boldsymbol{z}_u^c, \boldsymbol{z}_u^s\}$, encompasses all components derived from $\boldsymbol{x}$ and $\boldsymbol{u}$.

Supplementary tables

Table S1. Comparison of methods related to CauFinder

Method	Category	Input Data	Core Approach	Primary Application
CauFinder (this paper)	Causal Regulators Identification + State Transition	Bulk/scRNA-seq + Discrete/Continuous State Variable	Do-calculus + Information Flow + Network Control	Identifying causal relations from a set of key regulators to cell-state transitions, and further assessing their controllability
Mogrify (Rackham et al. [17])	Cell Conversion Factor Prediction	Gene expression data from defined source and target cell types + regulatory networks	Differential expression + transcription factor regulatory influence + redundancy reduction	Predicting transcription factors required for direct cell conversion between source and target cell types
LiNGAM (Shimizu et al. [6])	Causal Discovery	Bulk/scRNA-seq	Linear non-Gaussian acyclic model for causal ordering and directed effect estimation	Inferring causal ordering and directed effects under linear non-Gaussian assumptions
NOTEARS (Zheng et al. [4-5])	Causal Discovery	Bulk/scRNA-seq	Continuous optimization for DAG learning with a differentiable acyclicity constraint	Inferring directed causal graph structures; can be adapted to rank features connected to the state variable
FCI (Spirtes et al. [5])	Causal Discovery with Latent Confounders	Bulk/scRNA-seq	Constraint-based causal discovery using conditional independence tests, allowing latent confounders	Inferring partial ancestral causal structures under potential unobserved confounding
GES (Chickering et al. [7])	Score-based Causal Discovery	Bulk/scRNA-seq	Greedy search over equivalence classes of DAGs using a decomposable score	Inferring causal graph structures through score-based model selection
GENIE3 (Huynh-Thu et al. [13])	GRN Inference	Bulk/scRNA-seq	Tree-based Feature Selection (Random Forest/Extra-Trees)	Inferring relations from each gene to another (GRN) by ranking regulatory influences using tree-based models
Velorama (Singh et al. [14])	Causal GRN Inference	scRNA-seq + Pseudotime/RNA Velocity	Graph-based Granger Causal Inference (DAG) + TF Regulatory Speed Estimation	Inferring causal TF-target interactions & distinguishing fast/slow regulators in differentiation & disease
SCODE (Matsumoto et al. [16])	GRN Inference	scRNA-seq + Pseudotime	ODE-based GRN inference with matrix factorization	Inferring time-dependent relations from each gene to another (GRN) from

Method	Category	Input Data	Core Approach	Primary Application
				single-cell differentiation data
PIDC (Chan et al. [21])	GRN Inference	scRNA-seq	Partial Information Decomposition (PID) with Multivariate Information Theory	Inferring direct/undirected relations from each gene to another (GRN) from single-cell transcriptomic data by capturing higher-order statistical dependencies
CellOracle (Kamimoto et al. [10])	TF Perturbation Simulation	scRNA-seq + ATAC-seq	GRN refinement using TF motifs + Regularized Regression + Signal Propagation	Predicting TF effects and perturbation outcomes in GRNs
Aygun et al. [22]	Genetic Causal Inference	caQTL + eQTL + scRNA-seq	Bayesian Mediation Analysis (bmediatR) to infer QTL-driven regulatory pathways	Identifying cell-type-specific causal regulatory pathways linking genetic variants, chromatin accessibility, and gene expression
BEELINE (Pratapa et al. [23])	GRN Inference Benchmarking	scRNA-seq	Standardized pipeline to compare GRN inference methods	Benchmarking and comparing the performance of different GRN inference approaches

Legend. This table compares different methods with CauFinder, covering causal discovery, cell conversion factor prediction, gene regulatory network (GRN) inference, perturbation modeling, and benchmarking approaches. GRN refers to Gene Regulatory Networks, while TF stands for Transcription Factor. caQTL represents Chromatin accessibility Quantitative Trait Loci, and eQTL refers to Expression Quantitative Trait Loci. RNA velocity is a computational approach that predicts the future transcriptional state of a cell by analyzing the ratio of spliced and unspliced mRNA from single-cell RNA sequencing data.

Table S2. Top30 causal drivers from the drug response pairs

Gene	Causal Weight	TF
**SLC1A5**	0.00220	FALSE
**BARD1**	0.00217	FALSE
**CD9**	0.00209	FALSE
**DAAM1**	0.00207	FALSE
**TUBA1B**	0.00184	FALSE
**MSH6**	0.00166	FALSE
**TFDP1**	0.00137	TRUE
**ANXA2**	0.00134	FALSE
**RAB31**	0.00115	FALSE
**PLK2**	0.00113	FALSE
**GTF2B**	0.00103	TRUE
**MYO1B**	0.00102	FALSE
**DGKE**	0.000973	FALSE
**PSMC4**	0.000921	FALSE
**CD24**	0.000839	FALSE
**TGM2**	0.000742	FALSE
**HSPA2**	0.000700	FALSE
**RRBP1**	0.000681	FALSE
**TUBB4B**	0.000638	FALSE
**CEBPD**	0.000612	TRUE
**LMO7**	0.000604	FALSE
**TRIB1**	0.000475	FALSE
**HIST1H1C**	0.000380	FALSE
**KLF5**	0.000362	TRUE
**TPM1**	0.000334	FALSE
**TFAP2C**	0.000240	TRUE

SI References

[1] L. Kovačević, I. Newsham, S. Mukherjee, J. Whittaker, *arXiv preprint arXiv:2407.06015* **2024**.
[2] S. Shimizu, *Behaviormetrika* **2014**, *41* (1), 65.
[3] M. Kalisch, P. Bühlman, *Journal of Machine Learning Research* **2007**, *8* (3).
[4] X. Zheng, B. Aragam, P. K. Ravikumar, E. P. Xing, *Advances in neural information processing systems* **2018**, *31*.
[5] P. L. Spirtes, C. Meek, T. S. Richardson, *arXiv preprint arXiv:1302.4983* **2013**.
[6] S. Shimizu, T. Inazumi, Y. Sogawa, A. Hyvarinen, Y. Kawahara, T. Washio, P. O. Hoyer, K. Bollen, P. Hoyer, *Journal of Machine Learning Research-JMLR* **2011**, *12* (Apr), 1225.
[7] D. M. Chickering, *Journal of machine learning research* **2002**, *3* (Nov), 507.
[8] A. Paszke, S. Gross, F. Massa, A. Lerer, J. Bradbury, G. Chanan, T. Killeen, Z. Lin, N. Gimelshein, L. Antiga, *Advances in neural information processing systems* **2019**, *32*.
[9] F. Pedregosa, G. Varoquaux, A. Gramfort, V. Michel, B. Thirion, O. Grisel, M. Blondel, P. Prettenhofer, R. Weiss, V. Dubourg, *the Journal of machine Learning research* **2011**, *12*, 2825.
[10] K. Kamimoto, B. Stringa, C. M. Hoffmann, K. Jindal, L. Solnica-Krezel, S. A. Morris, *Nature* **2023**, *614* (7949), 742.
[11] P. Wang, X. Wen, H. Li, P. Lang, S. Li, Y. Lei, H. Shu, L. Gao, D. Zhao, J. Zeng, *Nature Communications* **2023**, *14* (1), 8459, https://doi.org/10.1038/s41467-023-44103-3.
[12] X. Cheng, M. Amanullah, W. Liu, Y. Liu, X. Pan, H. Zhang, H. Xu, P. Liu, Y. Lu, *Bioinformatics* **2023**, *39* (2), https://doi.org/10.1093/bioinformatics/btad071.
[13] V. A. Huynh-Thu, A. Irrthum, L. Wehenkel, P. Geurts, *PLoS One* **2010**, *5* (9), https://doi.org/10.1371/journal.pone.0012776.
[14] R. Singh, A. P. Wu, A. Mudide, B. Berger, *Cell Systems* **2024**, *15* (5), 462.
[15] V. Bergen, M. Lange, S. Peidli, F. A. Wolf, F. J. Theis, *Nature Biotechnology* **2020**, *38* (12), 1408, https://doi.org/10.1038/s41587-020-0591-3.
[16] H. Matsumoto, H. Kiryu, C. Furusawa, M. S. H. Ko, S. B. H. Ko, N. Gouda, T. Hayashi, I. Nikaido, *Bioinformatics* **2017**, *33* (15), 2314, https://doi.org/10.1093/bioinformatics/btx194.
[17] O. J. L. Rackham, J. Firas, H. Fang, M. E. Oates, M. L. Holmes, A. S. Knaupp, H. Suzuki, C. M. Nefzger, C. O. Daub, J. W. Shin, E. Petretto, A. R. R. Forrest, Y. Hayashizaki, J. M. Polo, J. Gough, F. C. The, *Nature Genetics* **2016**, *48* (3), 331, https://doi.org/10.1038/ng.3487.
[18] F. Wolf, P. Angerer, *Genome Biology* *19*, 15.
[19] a) S. Tang, Y. Xue, Z. Qin, Z. Fang, Y. Sun, C. Yuan, Y. Pan, Y. Zhao, X. Tong, J. Zhang, H. Huang, Y. Chen, L. Hu, D. Huang, R. Wang, W. Zou, Y. Li, R. K. Thomas, A. Ventura, K. K. Wong, H. Chen, L. Chen, H. Ji, *Natl Sci Rev* **2023**, *10* (4), nwad028, https://doi.org/10.1093/nsr/nwad028; b) Z. Fang, X. Han, Y. Chen, X. Tong, Y. Xue, S. Yao, S. Tang, Y. Pan, Y. Sun, X. Wang, Y. Jin, H. Chen, L. Hu, L. Hui, L. Li, L. Chen, H. Ji, *Signal Transduct Target Ther* **2023**, *8* (1), 16, https://doi.org/10.1038/s41392-022-01227-0.
[20] Y. Oren, M. Tsabar, M. S. Cuoco, L. Amir-Zilberstein, H. F. Cabanos, J.-C. Hütter, B. Hu, P. I. Thakore, M. Tabaka, C. P. Fulco, *Nature* **2021**, *596* (7873), 576.
[21] T. E. Chan, M. P. H. Stumpf, A. C. Babtie, *Cell Syst* **2017**, *5* (3), 251, https://doi.org/10.1016/j.cels.2017.08.014.
[22] N. Aygün, D. Liang, W. L. Crouse, G. R. Keele, M. I. Love, J. L. Stein, *Genome Biol* **2023**, *24* (1), 130, https://doi.org/10.1186/s13059-023-02959-0.
[23] A. Pratapa, A. P. Jalihal, J. N. Law, A. Bharadwaj, T. M. Murali, *Nat Methods* **2020**, *17* (2), 147, https://doi.org/10.1038/s41592-019-0690-6.
